# Genomic determinants of organohalide-respiration in *Geobacter lovleyi*, an unusual member of the *Geobacteraceae*

**DOI:** 10.1186/1471-2164-13-200

**Published:** 2012-05-22

**Authors:** Darlene D Wagner, Laura A Hug, Janet K Hatt, Melissa R Spitzmiller, Elizabeth Padilla-Crespo, Kirsti M Ritalahti, Elizabeth A Edwards, Konstantinos T Konstantinidis, Frank E Löffler

**Affiliations:** 1 School of Biology, Georgia Institute of Technology, 310 Ferst Drive, Atlanta, GA, 30332, USA; 2 Department of Cell and Systems Biology, University of Toronto, 25 Harbord Street, Toronto, ON, M5S 3G5, Canada; 3 School of Civil and Environmental Engineering, Georgia Institute of Technology, 311 Ferst Drive, Atlanta, GA, 30332, USA; 4 Department of Microbiology, University of Tennessee, M409 Walters Life Science Building, 1414 Cumberland Avenue, Knoxville, TN, 37996, USA; 5 Department of Chemical Engineering and Applied Chemistry, University of Toronto, 200 College Street, Toronto, ON, M5S 3E5, Canada; 6 Biosciences Division, Oak Ridge National Laboratory, P.O. Box 2008, Bethel Valley Road, Building 1520, Oak Ridge, TN, 37831, USA; 7 Department of Civil and Environmental Engineering, University of Tennessee, 223 Perkins Hall, Knoxville, TN, 37996, USA

## Abstract

**Background:**

*Geobacter lovleyi* is a unique member of the *Geobacteraceae* because strains of this species share the ability to couple tetrachloroethene (PCE) reductive dechlorination to *cis*-1,2-dichloroethene (*cis*-DCE) with energy conservation and growth (i.e., organohalide respiration). Strain SZ also reduces U(VI) to U(IV) and contributes to uranium immobilization, making *G. lovleyi* relevant for bioremediation at sites impacted with chlorinated ethenes and radionuclides. *G. lovleyi* is the only fully sequenced representative of this distinct *Geobacter* clade, and comparative genome analyses identified genetic elements associated with organohalide respiration and elucidated genome features that distinguish strain SZ from other members of the *Geobacteraceae*.

**Results:**

Sequencing the *G. lovleyi* strain SZ genome revealed a 3.9 Mbp chromosome with 54.7% GC content (i.e., the percent of the total guanines (Gs) and cytosines (Cs) among the four bases within the genome), and average amino acid identities of 53–56% compared to other sequenced *Geobacter* spp. Sequencing also revealed the presence of a 77 kbp plasmid, pSZ77 (53.0% GC), with nearly half of its encoded genes corresponding to chromosomal homologs in other *Geobacteraceae* genomes. Among these chromosome-derived features, pSZ77 encodes 15 out of the 24 genes required for *de novo* cobalamin biosynthesis, a required cofactor for organohalide respiration. A plasmid with 99% sequence identity to pSZ77 was subsequently detected in the PCE-dechlorinating *G. lovleyi* strain KB-1 present in the PCE-to-ethene-dechlorinating consortium KB-1. Additional PCE-to-*cis*-DCE-dechlorinating *G. lovleyi* strains obtained from the PCE-contaminated Fort Lewis, WA, site did not carry a plasmid indicating that pSZ77 is not a requirement (marker) for PCE respiration within this species. Chromosomal genomic islands found within the *G. lovleyi* strain SZ genome encode two reductive dehalogenase (RDase) homologs and a putative conjugative pilus system. Despite the loss of many *c*-type cytochrome and oxidative-stress-responsive genes, strain SZ retained the majority of *Geobacter* core metabolic capabilities, including U(VI) respiration.

**Conclusions:**

Gene acquisitions have expanded strain SZ’s respiratory capabilities to include PCE and TCE as electron acceptors. Respiratory processes core to the *Geobacter* genus, such as metal reduction, were retained despite a substantially reduced number of *c*-type cytochrome genes. pSZ77 is stably maintained within its host strains SZ and KB-1, likely because the replicon carries essential genes including genes involved in cobalamin biosynthesis and possibly corrinoid transport. Lateral acquisition of the plasmid replicon and the RDase genomic island represent unique genome features of the PCE-respiring *G. lovleyi* strains SZ and KB-1, and at least the latter signifies adaptation to PCE contamination.

## Background

*Geobacter* spp. are common members of anoxic freshwater sediment and subsurface microbial communities, where they are involved in the reduction of oxidized metal species and the turnover of organic matter [1]. Members of this genus show promise for bioremediation of anoxic subsurface environments contaminated with toxic radionuclides [2]. While dissimilatory metal reduction is a hallmark feature of *Geobacter*, the ability to use chlorinated organic compounds as electron acceptors has only recently been discovered in this genus, and appears to be restricted to a single *Geobacter* clade (Additional file 1) with only a few cultured representatives [3]. Organohalide respiration has been described for *Geobacter thiogenes* strain K and *Geobacter lovleyi* strain SZ, which dechlorinate trichloroacetate to dichloroacetate and tetrachloroethene (PCE) to *cis*-1,2-dichloroethene (*cis-*DCE), respectively [3,4]. In addition, another PCE-to-*cis-*DCE-dechlorinating *G. lovleyi* strain, designated strain KB-1, was identified in the PCE-to-ethene-dechlorinating consortium KB-1 [5]. *G. lovleyi* 16S rRNA gene sequences have been detected at the contaminated Oak Ridge IFRC site [6] and in trichloroethene (TCE)-contaminated sediments from Ft. Lewis, WA [7]. A recent continuous flow column study using the PCE-to-ethene-dechlorinating bioaugmentation consortium Bio-Dechlor INOCULUM (BDI) containing *G. lovleyi* strain SZ indicated that PCE-dechlorinating *Geobacter* strains enhance dissolution of free phase PCE [8]. Further, *G. lovleyi* strain SZ uses graphite electrodes as a direct electron donor for reductive dechlorination (organohalide respiration), possibly enabling innovative bioremediation approaches [9]. The organohalide-respiring *Geobacter* strains share 16S rRNA gene sequences with 98–100% identity to each other but only 93% identity with *G. sulfurreducens* strain PCA, the type strain for the *Geobacter* genus. Among the *δ-ProteobacteriaDesulfuromonas michiganensis* is the only other PCE-to-*cis*-DCE-respiring species but genome information is not available [10]. The genus *Anaeromyxobacter* (*δ-Proteobacteria*) comprises isolates with sequenced genomes, which are capable of using chlorinated phenols as respiratory electron acceptors, but PCE dechlorination has not been reported [11-13].

Due to its implications for bioremediation of sites contaminated with both chlorinated ethenes and radionuclides [3], and the paucity of genome information of PCE-respiring *δ-ProteobacteriaG. lovleyi* strain SZ is a promising reference strain for understanding the physiological and evolutionary responses of microbes to anthropogenic changes in the environment. Here we present the *G. lovleyi* strain SZ genome sequence, including the discovery of a unique 77 kbp plasmid (pSZ77). The plasmid is notable, as it contains laterally acquired genes and some that encode functions important to *Geobacter* metabolism such as cobalamin biosynthesis. Comparative analyses of the strain SZ genome revealed significant functional divergence from previously sequenced *Geobacter* spp. and mobile elements sharing genomic features of *Pelobacter*, a distinct genus of the *δ-Proteobacteria*.

## Methods

### Cultures and growth conditions

*G. lovleyi* strain SZ [3], *G. lovleyi* strain KB-1 (AY780563) [5], *G. thiogenes*[4], and four *G. lovleyi* isolates Geo7.1, Geo7.2, Geo7.3, and Geo7.4 obtained from PCE-to-*cis*-DCE-dechlorinating microcosms established with Fort Lewis, WA, soil [14] were utilized in this study. *G. lovleyi* strain KB-1 was isolated from consortium KB-1. Following a series of eight 1:20 vol/vol dilution transfers to defined mineral medium supplemented with 90 mg L^−1^ PCE and 10 mM acetate, the final dilution culture was used to inoculate a set of serial dilution agarose shake tubes amended with 10 mM fumarate and 10 mM acetate [15]. Transfer of a colony from a 10^−2^ dilution tube to liquid culture yielded a pure culture of *G. lovleyi* strain KB-1. In addition, the PCE-to-ethene-dechlorinating consortia BDI and KB-1 [5] were grown and maintained with 0.05 mg mL^−1^ PCE as electron acceptor. Pure and mixed cultures were grown in 60 mL serum bottles containing 40 mL reduced, defined mineral salts medium with 5 mM acetate serving as both carbon source and electron donor with an N_2_-CO_2_ (80:20, vol/vol) headspace [16]. Lactate (5 mM) substituted for acetate to grow *G. thiogenes* and consortium BDI. The KB-1 consortium was maintained with methanol as an electron donor, amended to 5 times the electron equivalents required for complete dechlorination. To test the effects of cyanocobalamin (CN-Cbl) on PCE dechlorination, cultures of strain SZ were amended with 0.05 mg mL^−1^ PCE as the sole electron acceptor and 0, 15, and 750 μg CN-Cbl L^−1^. Chlorinated ethenes were quantified by gas chromatography as described [17,18].

### Plasmid stability

To explore if plasmid pSZ77 was stably maintained (i.e., segregational stability), strain SZ cultures were grown at 35°C with 20 mM sodium fumarate as the electron acceptor in medium with 1,500 μg L^−1^ and without CN-Cbl. When visible turbidity was apparent after 24 to 72 hours, the cultures were consecutively transferred (1% inoculum size, vol/vol) for at least 20 times to fresh medium. Plasmid curing was also attempted by amending liquid cultures with 0.001–0.1% SDS (w/vol) [19] or 0.1 M L-ascorbic acid [20].

### Genomic DNA extraction and PCR

PCR primers were designed using Primer3 software (http://frodo.wi.mit.edu) based on the predicted open reading frame (ORF) encoding the homolog to the replication initiation protein RepA identified on pSZ77. The primers repA_136F (5'-AGCATCGGTCAGCTGAATCT-3'), and repA_700R (5'-GGTTAGAGCGTGGTGCATTT-3') were used to amplify a 565 bp fragment of the pSZ77 *repA* gene. To test for the presence of *repA* in other cultures, biomass was collected from 2 mL aliquots of pure and mixed cultures by centrifugation at 13,200 rpm for 20 min at room temperature (RT). The pellet was added to a bead tube and the DNA extracted according to the protocol for the PowerSoil DNA isolation kit (Mo Bio Laboratories, Inc.) provided by the manufacturer. PCR reactions were prepared in a volume of 15 μL containing 1x PCR buffer, 1.5 mM MgCl_2_, 200 μM dNTPs, 200 nM of each forward and reverse primers, 0.02 U of GoTaq DNA polymerase and 1 μL of DNA template. The PCR thermocycler program for the *repA*-targeted primers was 94°C for 2 min, followed by 30 cycles of denaturation at 94°C for 30 s, annealing at 56°C for 30s, extension at 72°C for 30s, and a final extension at 72°C for 6 min. PCR was also carried out using the primer pair Geo564F/840R [21] that targets the 16S rRNA gene of all *Geobacteraceae*. PCR conditions and the amplification profile used for the Geo564F/840R set were as described for the *repA* primer set.

### Plasmid isolation

For large plasmid isolation using a modified protocol based on the Kieser method [22,23], 50 to 200 mL of fumarate-grown SZ culture and 100 mL each of fumarate-grown *G. thiogenes* and the four Fort Lewis *Geobacter* isolates were collected by centrifugation (3,220 x *g*, 30 min). The DNA was isolated as previously described except that the plasmid DNA was precipitated by standard methods [24] and suspended in a final volume of 50–100 μL TE buffer (10 mM Tris, 1 mM EDTA [pH 8.0]). Plasmid DNA extracts were separated by electrophoresis through a 0.7% (w/vol) agarose Tris-acetate gel run in 1x Tris-acetate EDTA (TAE) buffer [24]. DNA was visualized by staining in 0.5 μg ethidium bromide per mL TAE buffer solution. Isolate *G. lovleyi* strain KB-1 was grown with 10 mM acetate and 90 mg L^−1^ PCE, and plasmid DNA was extracted from biomass obtained from 1 L of culture suspension using the Qiagen Plasmid Midi Kit and the modified protocol for large inserts (http://www.qiagen.com/literature/handbooks/default.asp).

### Sequencing

Genomic DNA of *G. lovleyi* strain SZ was extracted from cells grown with acetate and fumarate following established protocols (Bacterial genomic DNA isolation using CTAB. http://my.jgi.doe.gov/general/). Sequencing was performed by the Department of Energy’s Joint Genome Institute (JGI) using a combination of 454 and Sanger reads with average lengths of 107 and 949 bp, respectively. The total 662,511 reads provided an average 25-fold coverage for the chromosome and 40-fold coverage for the plasmid. The *G. lovleyi* strain SZ genome sequences have been assigned GenBank accession numbers CP001089 and CP001090 for the chromosome and pSZ77, respectively. DNA was extracted from consortium KB-1 following an established protocol [25], and clone libraries were generated by the JGI using in-house protocols (http://www.jgi.doe.gov/sequencing/protocols) and sequenced by the Sanger approach. The strain KB-1 plasmid DNA was incorporated into a bar-coded 454 GS FLX Titanium sequencing run at the Center for Applied Genomics at the University of Toronto. The 454 reads, along with the consortium KB-1 metagenome contigs identified as *Geobacter* plasmid sequences, were aligned against pSZ77 using the Geneious assembly tool (http://www.geneious.com). Metagenome reads with disagreements with the pSZ77 sequence were verified and removed if necessary. Read depth was at least 3-fold for most of the assembly with a maximum of 12-fold coverage, but as low as single coverage in two regions of less than 136 bp. Amplicons of the 16S rRNA genes of the Ft. Lewis *G. lovleyi* strains Geo7.1, Geo7.2, Geo7.3, and Geo7.4 were sequenced twice each by the Sanger approach and assigned the GenBank accession numbers JN982204 through JN982211.

### Computational analyses

Customized Perl scripts were used to determine GC percentage of all predicted protein-coding genes on the strain SZ chromosome and pSZ77 with standard deviations from genomic averages computed in R (http://www.r-project.org/). COGs assignments were determined from the NCBI .ptt files for the *Geobacteraceae* and related non-*Geobacteraceae* chromosomes (AE017180, CP000148, CP000698, CP001390, CP001124, CP001661, CP002479, CP000482, and CP000142) and plasmids (CP000149 CP000483, and CP000484) using customized Perl scripts. Candidate *c*-type cytochromes were determined by searching all strain SZ amino acid sequences for the CxxCH motif using a customized Perl script, and then further screened for homology to *c*-type cytochromes in the RefSeq database using PSI-Blast (e-value = 1e-11, h = 1e-5) [26] and the PROSITE profile for *c*-type cytochromes (ca.expasy.org/tools/scanprosite/). CxxCH-containing sequences lacking over 50% of PSI-Blast matches annotated as *c*-type cytochromes or lacking recognizable *c*-type cytochrome PROSITE profiles (i.e., PS51007, PS51008, PS51009, and PS51010) were eliminated from further analysis. Trans-membrane regions of predicted outer membrane receptor proteins were determined using Pred-TMBB [27]. The nucleotide sequence of pSZ77 and the chromosomal genomic islands on the SZ chromosome were analyzed for repeats using REPuter [28]. Codon adaptation indices (CAI) for strain SZ ORFs encoded on the plasmid and chromosomal genomic islands were computed against a codon usage table based upon all strain SZ chromosomal ORFs using the E-CAI server [29]. Computed CAI were normalized to the expected CAI based upon a 5% level of significance for the bootstrapped set of all ORFs on the SZ chromosome, such that putative foreign genes (i.e. non-*Geobacter*) would score < 1.00 [29]. The origin of replication of pSZ77 was identified using Ori-Finder [30], which searches for DnaA-binding sites at regions of GC-skew reversals. The amino acid sequences of replication initiation genes (*repA*) were used to infer plasmid phylogeny and 16S rRNA genes DNA sequences were used to infer genome phylogeny. All alignments were performed in MUSCLE [31] and used to build trees in Phylip with topology inferred by bootstrapped neighbor-joining and branch lengths computed by maximum likelihood [32]. 16S rRNA gene sequences were also used to confirm family and genus affiliation using the Ribosomal Database Project [33]. Trees were visualized and formatted using the interactive Tree of Life tool [34].

Translations of all predicted GenBank ORFs on pSZ77 were run through BlastP [35] against the nr database with BLOSUM45 and an e-value cutoff of 0.00001. To determine pSZ77 genes and chromosomal genomic islands acquired from bacteria outside of the *Geobacteraceae*, all translated ORFs on pSZ77 and the SZ chromosome were queried using BlastP against the genomes of *Geobacter sulfurreducens* strain PCA (AE017180), *Geobacter metallireducens* strain GS-15 (CP000148), *Geobacter uraniireducens* strain Rf4 (CP000698), *Geobacter* sp. strain FRC-32 (CP001390), *Geobacter bemidjiensis* strain Bem (CP001124), *Geobacter* sp. strain M21 (CP001661), *Geobacter* sp. strain M18 (CP002479), *Pelobacter propionicus* DSM 2379 (CP000482), and *P. carbinolicus* DSM 2380 (CP000142). Pairwise average amino acid identities between predicted proteomes were mined from BlastP output using custom Perl scripts and genome affiliations of top BlastP matches were checked against IMG statistics for *G. lovleyi* strain SZ (img.jgi.doe.gov). Sequence similarities to orthologs for pSZ77 cobalamin biosynthesis genes, pSZ77 *repA*, the chromosomal *pce*-genes, and chromosomal conjugative pilus *tra*-genes were determined using PSI-Blast [26]. Further functional inference of amino acid sequences from plasmid-or genomic-island-encoded genes were performed by parsing for conserved domains in PFAM (pfam.janelia.org) and conserved motifs in PROSITE (ca.expasy.org/tools/scanprosite/). A physical map of pSZ77 was plotted using CGview [36].

## Results and discussion

### The *Geobacter lovleyi* strain SZ genome

Sequencing of the *G. lovleyi* strain SZ genome revealed both typical *Geobacteraceae* characteristics (e.g., genes encoding multiheme *c*-type cytochromes) and elements not previously found among members of the *Geobacter* genus, including genes encoding putative reductive dehalogenases (RDases) and a 77-kbp plasmid designated pSZ77. The 54.8% GC content, 3,644 predicted open reading frames (ORFs), and 3.9 Mb size of the strain SZ chromosome (Table 1) are comparable to other sequenced genomes of members of the *Geobacter* genus, *G. sulfurreducens* PCA [37], *G. metallireducens* GS-15 [38], *G. bemidjiensis* Bem [39], *G. uraniireducens* Rf4 [40], and *Geobacter* spp. strains FRC-32 [41], M21, and M18, all ranging from 3.8 to 5.1 Mb in size. The strain SZ chromosome has 279 chromosomal genes assigned to the energy production clusters of orthologous groups (COGs) (class C), a somewhat lower count compared to total COGs class C genes on the seven other sequenced *Geobacter* genomes (ranging from 280 to 356, avg. 334), indicating a shift in the strain SZ respiration-related gene repertoire. By comparison, the 481 strain SZ genes functionally classified in the signal transduction COGs (class T) lies within the range for *Geobacter* spp. (382–587, avg. 427). SZ chromosomal genes have 40% of their top BlastP matches in the genomes of other *Geobacter* spp. (Table 1), where average BlastP identities between the strain SZ and each *Geobacter* proteome range from 53 to 56%. By comparison, pSZ77 has 53.0% GC content and only eight (11%) of its 81 total predicted ORFs with top BlastP matches (avg. identity 77%) among *Geobacter* spp. (Table 2).

**Table 1:** **General features of the*Geobacter lovleyi*strain SZ chromosome**

**Chromosome**		
*General features*		
	Size (bp)	3,917,761
	GC percentage	54.8
	Total number of predicted protein-coding genes	3,644
	Number of pseudogenes	38
*Protein-coding genes*		
	Total intact protein-coding genes	3,606
	- matching annotated homologs	3,138
	- matching hypotheticals only	296
	- with no public database matches	171
	- in one or more COGs	2817
	- in energy production COGs	279
	- in coenzyme metabolism COGs	122
	- as predicted *c*-type cytochromes	49
	- percent with top BlastP match in *Geobacter* spp.	40%
	- percent with top BlastP match in *Pelobacter* spp.	29%
	- percent with top BlastP match in other *δ-Proteobacteria*	5%
*Non-protein-coding features*		
	Number of rRNA operons (16S-23S-5S)	2
	Number of transfer RNAs	45
	CRISPR regions	1

**Table 2:** **General features of the*G. lovleyi*strain SZ plasmid pSZ77**

**Plasmid**		
	Size (bp)	77,113
	GC percentage	53.0
	Total number of protein-coding genes	81
	Number of pseudogenes	2
*Protein-coding genes*		
	Number of intact protein-coding genes	79
	- matching annotated homologs	66
	- matching genes with hypothetical function only	6
	- with no public database matches	7
	- in one or more COGs	66
	- in energy production COGs	3
	- in coenzyme metabolism COGs	18
	- predicted as *c*-type cytochromes	0
	- percent with top BlastP match in *Geobacter* (7 genomes)	11%
	- percent with top BlastP match in *Pelobacter* (2 genomes)	24%
	- percent with top BlastP match in other *δ-Proteobacteria*	37%
*Non-protein-coding features*		
	Number of rRNA operons (16S-23S-5S)	0
	Number of transfer RNAs	0

### Organohalide respiration

**Figure 1:**
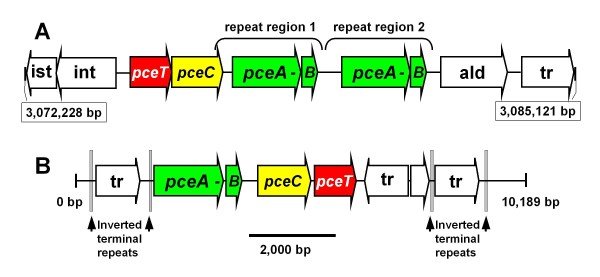
**PCE dehalogenase (Pce) genomic island on the*G. lovleyi*strain SZ chromosome (CP001089; Glov_2866-Glov_2875) (Panel A) compared to the transposon-encoded*pce*-gene cluster from*Desulfitobacterium hafniense*strain TCE1 (AJ439608) (Panel B).** The transposase-associated *pce*-gene clusters in the genomes of *D. hafniense* strain Y51 (AP008230) and *Dehalobacter restrictus* PER-K23 (AJ439607) share 99% nucleotide identity with the strain TCE1 *pce*-gene cluster. Inferred gene functions are abbreviated as follows: *pceA* - putative tetrachloroethene (PCE) dehalogenase, *pceB* - membrane anchor protein subunit, *pceT* - peptidylprolyl-*cis*-*trans*-isomerase, *pceC* - FMN-binding and polyferredoxin domain protein, int - integrase, ist - ATPase, tr‒transposase, and ald‒aldehyde dehydrogenase. The *pceA-pceB* ORFs are duplicated within the strain SZ genomic island, and associated with transposase genes, but no inverted repeats were detected. In contrast, the RDase genes in strain TCE1 are associated with both transposases and inverted repeats, and the overall structure of the element in TCE1 resembles a composite transposon.

A defining feature distinguishing the *G. lovleyi* strain SZ chromosome from other *Geobacter* genomes is the presence of a gene cluster related to organohalide respiration. The ability of *G. lovleyi* strains to respire PCE resides on a chromosomal genomic island (Figure 1A) containing two putative PceA RDases. The *pce*-genes predicted to play a role in PCE respiration, *pceT-pceC-pceA1-pceB1-pceA2-pceB2*, (Glov_2866-Glov_2875) comprise a region with a GC content of 37%, as much as 2.5 standard deviations below the chromosomal average of 54.8%. The Codon Adaptation Index (CAI), a comparative measure of codon usage, for the six *pce*-genes cluster fall below the average for SZ chromosomal ORFs (normalized CAI < 1.00; Additional file 2) indicating recent acquisition by the SZ genome. The ‘Pce’ chromosomal region, in which the *pce*-genes reside, exhibits an apparent reversal in GC-skew (Additional file 3). Finally, the six *pce*-genes have no homologs in any other *Geobacter* or related *Pelobacter* genomes. Together, these features indicate that the SZ chromosomal Pce region encoding the *pce*-genes in an atypical region acquired by lateral gene transfer.

The six *pce*-genes of the *G. lovleyi* genome are homologous to genes in functionally characterized PCE respiration gene clusters. Functions for three of the four components of the *pce*-gene cluster, *pceA-pceB-pceC-pceT*, from the *Firmicutes* (Figure 1B) is supported by experimental evidence in *Desulfitobacterium hafniense* strains TCE1 [42] and Y51 [43] and *Dehalobacter restrictus* strain PER-K23 [44]. *pceA* encodes the catalytic PceA RDase subunit, whose activity towards organohalides is dependent upon a bound cobalamin cofactor [44]. *pceB* is inferred to encode a membrane-bound subunit to PceA, but direct experimental evidence for this function is lacking. *pceC* is co-transcribed with *pceA-pceB* in *D. hafniense* Y51 and the PceC protein is believed to function in regulating *pceA* gene expression and electron transfer to the PceA protein [45]. *pceT* encodes a protein shown to function as a chaperone to the PceA preprotein [46]. The order of the predicted *G. lovleyi pce*-genes, *pceT-pceC-pceA1-pceB1-pceA2-pceB2*, differs from that of the functionally-characterized *pce*-gene clusters (Figure 1B). The *G. lovleyi pceA-pceB* ORFs, encoding the predicted PCE RDase catalytic subunit and the membrane anchor subunit, respectively, are duplicated on tandem 2,466 bp long blocks sharing 99.8% nucleotide identity (Figure 1A). Despite apparent differences in organization, the amino acid sequences encoded by all six *G. lovleyi pce*-genes have their most similar homologs among the functionally characterized *pce*-gene clusters from *Desulfitobacterium hafniense* strain Y51 (AB070709), *D. hafninese* strain TCE1 (AJ439608), and *D. restrictus* (AJ439607). Both sets of strain SZ’s putative PceA and PceB proteins share 33–36% amino acid (aa) identity with PceA (RDase A subunit, BAE84628) and PceB (RDase B subunit, BAE84627) from *D. hafniense* Y51 [43]. The *G. lovleyi* PceAs share only distant similarity with the genomic-island-encoded VcrA RDase of *Dehalococcoides* sp. strain VS (18% identity, 30% similarity) [47]. Glov_2869 (putative PceC) shares a FMN-binding domain (pfam04205), a polyferredoxin (COG0348) domain, and 33% aa identity with the *D. hafniense* Y51 PceC regulatory protein (BAE84626). Glov_2868 (putative PceT) is annotated as 'peptidylprolyl isomerase' and shares 19% aa identity with PceT from *D. hafniense* (BAE84625). Despite their apparent shared conserved functions, the low sequence similarities and lack of synteny between strain SZ and the *Desulfitobacterium* spp. *pce*-gene clusters suggests the gene clusters have diverged from their shared ancestral gene cluster over time.

An IS21-like integrase gene cluster and a transposase gene flank the strain SZ *pce*-genes upstream and downstream, respectively, but neither exhibits the compositional features suggestive of recent lateral gene transfer, with GC content, codon biases, and GC-skew consistent with the *G. lovleyi* genome. Transposase-associated repeats were found to mediate circularization, and presumably excision, of the *D. hafniense* strain TCE1 PceA-encoding transposon [42]. In contrast, no repeats flanking the SZ PceA genomic island could be detected, and the ISL3-superfamily transposase (pfam01610) downstream of the SZ *pce*-genes lacks detectable similarity with the mutator family transposases (pfam00872) found on the *D. hafniense* strain TCE1 transposable element. Furthermore, the *rve* family integrase (pfam00665) and IS21-type ATPase/integrase cluster upstream of the SZ *pce*-genes, along with adjacent non-coding DNA (Figure 1A), do not share homology with the *dsiB* integrase in the *vcrA* genomic islands of *Dehalococcoides*[48] nor any other mobilization-related genomic elements (inverted repeats, direct repeats, etc.) associated with RDase genes [49]. The integrase and the ATPase instead appear to originate from within the *Geobacter* genus, sharing 62% and 80% aa identity, respectively, with their homologs in *G. uraniireducens*. The SZ Pce genomic island integrase and transposase lack homology with known RDase-associated mobile elements, while the GC content of the *pce*-genes suggests much of this element was acquired by strain SZ relatively recently. The apparent divergence of the strain SZ *pce*-genes from their closest known orthologs in *Desulfitobacterium* spp. suggests the SZ genes are not a recent lateral transfer from the *Desulfitobacterium*/*Dehalobacter* group but from another, yet unidentified donor.

### F-factor conjugation

**Figure 2:**
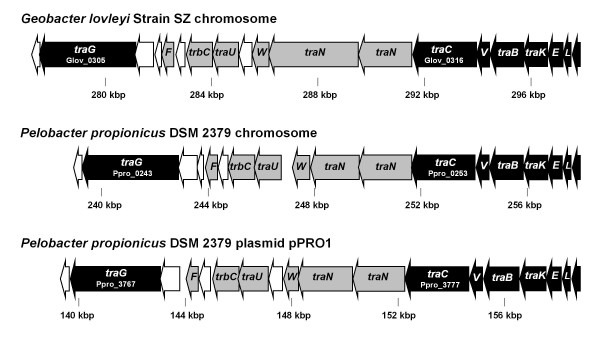
**The*G. lovleyi*strain SZ chromosomal genomic island encoding the putative F-type conjugative pilus*tra*-gene cluster (Glov_0304 to Glov_0322) and the orthologous gene clusters on the chromosome and 202 kbp plasmid pPRO1 of*P. propionicus.*** Black ORFs represent genes homologous to conjugative pilus cluster core genes, likely functioning in pore complex assembly and mating pair stabilization. Gray ORFs are homologous to F-type conjugative pilus accessory genes while unshaded ORFs lack inferred function.

A second predicted chromosomal genomic island harbors a predicted F-factor conjugative pilus *tra*-gene cluster (Glov_0304 - Glov_0322), *traE-L-E-K-B-V-C-N-N-W, traU-trbC, traF, traG,* (Figure 2), which lacks homologs in any sequenced *Geobacter* genome. The cluster of *tra*-genes form part of a region (‘Tr’ in Additional file 3) spanning 108 ORFs, flanked by a transposase (Glov_0226) and a resolvase (Glov_0336) gene. Aside from the 19 *tra*-genes, 20 of the Tr region genes are assigned to COGs class L or class V, which include endo/exoribonucleases and helicases, while another 42 genes have hypothetical or unknown functions. A total of 62 genes in the Tr region, including a majority of the *tra*-genes, share > 75% aa identity with orthologs on the chromosome of *Pelobacter propionicus* DSM 2380. Given that the average SZ chromosome-encoded protein shares 57% identity with BlastP matches in *P. propionicus*, the Tr region composition suggests lateral acquisition of this region, possibly from a *Pelobacteraceae*-like donor. The region is designated as a genomic island based on its similarity to a non-*Geobacteraceae* genome and the predominance of hypothetical genes and DNA-manipulating genes [50]. An alternate hypothesis for the origin of the Tr region is a shared ancestry within the order *Desulfuromonadales*, which includes the *Pelobacteraceae* and the *Geobacteraceae*, with subsequent loss of this element in the *Geobacter*. With only seven sequenced *Geobacter* genomes available, vertical inheritance cannot be ruled out; however, the high similarity to homologs in *Pelobacter* does suggest that the *tra*-gene cluster is part of a genomic island.

The *tra*-gene cluster, Glov_0304 to Glov_0322, encodes proteins homologous to known plasmid-encoded DNA-transfer F-type conjugative pili [51]. Out of the 19 proteins encoded on the *tra*-gene cluster, five have PSI-Blast matches to genes unique to F-type conjugative pili (i.e., *traNtraW*, and *traU*), while seven have PSI-Blast matches to conserved F-type and P-type conjugative pilus “core” genes: *traEtraLtraKtraBtraVtraC*, and *traG*[51,52] (Figure 2). All but one of the proteins on the strain SZ putative F-type pilus cluster share synteny and high similarity with genes on the chromosome of *P. propionicus* (sharing 40–91% aa identity) and the 202 kbp plasmid pPRO1 of *P. propionicus* (37–61% aa identity) (Figure 2). Nearly half of the 19 genes in the strain SZ conjugative pilus cluster have GC contents near the strain SZ genomic average and normalized CAI > 1.00 (Additional file 4), suggesting a sufficient residence time on the SZ genome to ameliorate to SZ chromosomal codon usage [53]. Four additional genomic islands on the SZ chromosome are predicted based on low %GC, disruption of GC-skew, and/or the lack of homologs in a majority of other *Geobacter* spp. genomes. These additional inferred genomic islands, hyp1, hyp2, and hyp3 encode hypothetical proteins and proteins assigned to COGs class L or class V, while genomic island M contains five genes predicted to function in capsule polysaccharide biosynthesis (Additional file 3).

### Electron transfer and oxidoreductases

Additional features distinguishing the *G. lovleyi* strain SZ genome from other *Geobacteraceae* include the reduced number of genes encoding *c*-type cytochromes and the lack of several key genes related to oxygen tolerance and reactive oxygen species (ROS) detoxification. The *G. lovleyi* strain SZ genome encodes 49 ORFs encoding *c*-type cytochromes (Table 3, Additional file 5), of which only six are predicted multi-heme cytochromes with 10 or more CxxCH heme-binding motifs, and none have more than 12 CxxCH motifs (Table 3). In contrast, described *Geobacter* genomes encode 76–104 *c*-type cytochromes, of which 17–31 have 10 or more CxxCH motifs, with a maximum of 43 heme-binding motifs [54]. *P. propionicus* has 20 ORFs predicted to encode *c*-type cytochromes, with only one having more than 10 heme-binding motifs (Table 3, Additional file 6).

**Table 3:** **Predicted energy metabolism genes identified on the genomes of*Geobacter*spp. and*Pelobacter propionicus***.

Genome	Protein-coding genes in energy production COGs (class C)	Number of predicted *c*-type cytochromes	*c*-type cytochromes with >10 heme-binding motifs
*G. lovleyi*	279	49	6
*G. sulfurreducens*	280	89 *	23
*G. metallireducens*	356	76 *	17
*G. uraniireducens*	355	104 *	31
*P. propionicus*	334	19	1

* According to Butler et al. [54].

For each genome, number of *c*-type cytochromes with more than 10 predicted heme-binding sites is positively correlated with total predicted *c*-type cytochromes (R = 0.98).

The reduced set of multi-heme *c*-type cytochromes in strain SZ (Table 3, Additional file 5) appears adequate in mediating respiration with U(VI), Mn(IV), and Fe(III) oxides [3] and electrode surfaces [9]. Specific functions have only been determined for cytochromes with 10 or fewer hemes [54]. Decaheme *c*-type cytochromes have been confirmed to mediate respiration on electrodes in *Shewanella*[55] and an 8-heme outer-membrane cytochrome has been shown to be involved in electron transfer to solid state electrodes in *Geobacter sulfurreducens*[56]. Smaller outer membrane *c*-type cytochromes with 4–6 hemes are essential for *Geobacter* respiration with insoluble Fe(III) and Mn(IV) oxides [57]. PilA-type pilins may play an essential role in metal reduction [58], particularly in radionuclide reduction [59]. Strain SZ encodes a putative PilA (Glov_2096) sharing 81% aa identity with the conductive pilus protein (PilA) of *G. sulfurreducens* (GSU1496) [58]. *P. propionicus* also encodes a putative PilA (Ppro_1656), which shares 80% identity with the *G. sulfurreducens* PilA. Yet, *P. propionicus* has a reduced repertoire of *c*-type cytochromes, and is completely lacking decaheme *c*-type cytochromes (Additional file 6). Accordingly, *P. propionicus* lacks the ability to respire on electrodes or radionuclides [60]. By contrast, the strain SZ genome carries the predicted minimal set of genes to allow utilization of a comparable range of electron acceptors as other *Geobacter* spp. [3].

Additional genes that are key to *Geobacter* electron transfer are present as multiple paralogs on the strain SZ genome. The strain SZ genome encodes 11 molybdopterin oxidoreductase domain proteins (pfam00384), a larger number compared to other *Geobacter* genomes. The strain SZ molybdopterin oxidoreductase-type proteins, for which function can be inferred, include three nitrate reductases and two formate dehydrogenases (Additional file 7). One of the nitrate reductase proteins is a periplasmic-type, sharing 43% aa identity (61% similarity) with the respiratory nitrate reductase (NapA) from *Desulfovibrio desulfuricans*[61]. The role of the two predicted formate dehydrogenases is unclear, as strain SZ does not utilize formate as an electron source or carbon source under PCE- or Fe(III)-reducing conditions [3]. The SZ chromosome encodes seven fumarate reductase/succinate dehydrogenase-type flavoproteins (pfam00890), while no other *Geobacter* genome encodes more than three. Strain SZ uses fumarate as an electron acceptor and has the full set of genes encoding TCA cycle enzymes, but it is not clear which of these fumarate reductases play a role in these pathways. Strain SZ has five gene clusters (one on pSZ77) encoding pyruvate ferredoxin/flavodoxin oxidoreductase (PFOR) complexes (Additional file 7) of the type inferred to mediate incorporation of acetate into the TCA cycle in *G. sulfurreducens*[62]. Strain SZ lacks any homologs to subunit A of the pyruvate dehydrogenase E1 complex, making it unclear how the organism is able to use pyruvate as an electron donor [3].

With regard to ROS and oxygen detoxification, strain SZ lacks homologs to the heme-copper (*aa3*) cytochrome *c* oxidases involved in the utilization of oxygen in *G. sulfurreducens*[63]. Accordingly, strain SZ does not respire oxygen [3] and responds negatively to *in situ* oxygen exposure [12]. Strain SZ possesses a complete gene cluster encoding homologs to the cytochrome *bd* ubiquinol oxidase complex shown to mediate oxygen tolerance in anaerobes (Glov_1208-Glov_1209, Additional file 8) [64], but lacks any homolog to the diheme cytochrome *c* peroxidase (MauG) or superoxide dismutase (SodA) shown to be strongly expressed in the presence of oxygen in *G. uraniireducens*[65]. *P. propionicus* similarly lacks heme-copper (*aa3*) cytochrome *c* oxidase, MauG, and SodA. The absence of multi-heme *c*-type cytochromes with more than 12 hemes in strain SZ may reflect differences in habitat and metabolic strategy as compared to other *Geobacter* spp. The high-molecular weight *c*-type cytochromes with 13 or more hemes in *G. sulfurreducens* were suggested to function as biological capacitors [66], providing storage for reducing equivalents while the energy-stressed *Geobacter* cell is seeking new sources of terminal electron acceptors. Although this is an intriguing hypothesis, the synthesis of such high molecular weight *c*-type cytochromes under nutrient limitations burdens the cell’s energy budget, and the true benefit for survival has yet to be demonstrated. The poor oxygen tolerance exhibited by strain SZ, confining it to persistently anoxic habitats, may be associated with the reduced count of high molecular weight, multi-heme *c*-type cytochromes prevalent in aerotolerant *Geobacter*. Strain SZ may utilize a strategy for temporary electron storage that is not dependent on *c*-type cytochromes or is able to employ cytochromes with fewer hemes to the same effect seen in aerotolerant *Geobacter*.

Consistent with all other *Geobacteraceae* genomes, strain SZ possesses the full set of genes for the *de novo* biosynthesis of both heme and cobalamin (Table 4). Yet, unlike any other *Geobacter* spp. or *Pelobacter* spp. genome, a majority of cobalamin biosynthesis genes in the SZ genome are localized to a plasmid.

**Table 4:** **Predicted heme and cobalamin biosynthesis genes on the*G. lovleyi*strain SZ genome in comparison to the model organism for anaerobic cobalamin biosynthesis,*Salmonella enterica*subsp.*enterica*ser. Typhimurium**

	*G. lovleyi* SZ genome		Homolog in *Salmonella enterica* subsp. *enterica* ser. Typhimurium LT2		
	Chromosome	Plasmid pSZ77	Gene symbol	Function	% Ident. (%Simil.)
					Chr.^#^pSZ77
**Early heme biosynthesis**	Glov_0501		*hemD*	Uroporphyrinogen-III synthase	no match
	Glov_0502		*hemC*	Porphobilinogen deaminase	52(65)
	Glov_0503		*hemA*	Glutamyl-tRNA reductase	40(62)
	Glov_0688		*hemB*	δ-Aminolevulinic acid dehydratase	47(63)
**Late heme biosynthesis**	Glov_0521		*hemY*	Protoporphyrinogen oxidase	no match
	Glov_1228		*hemN*	Coproporphyrinogen III oxidase	27(45)
	Glov_2930		*hemH*	Ferrochelatase	31(47)
	Glov_3245		*hemL*	Uroporphyrinogen decarboxylase	52(67)
**Cobalamin biosynthesis**	Glov_3417	Glov_3646	*cbiE*	Precorrin-6y C5,15-methyl-transferase	32(54)35(57)
		Glov_3647	*cbiJ*	Precorrin-6x reductase	30(44)
		Glov_3648	*cbiH*	Precorrin-3B C17-methyltransferase	46(63)
		Glov_3649	*cbiG*	Cobalamin biosynthesis protein	31(49)
		Glov_3650	*cbiF*	Precorrin-4 C11-methyltransferase	50(68)
		Glov_3651	*cbiD*	Cobalamin biosynthesis protein	35(51)
		Glov_3652	*cbiL*	Precorrin-2 C20-methyltransferase	33(52)
		Glov_3653	*cbiK*	Anaerobic cobalt chelatase	32(45)
		Glov_3654	*cbiC*	Precorrin-8X methylmutase	34(52)
		Glov_3655	*cbiA*	Cobyrinic acid a,c-diamide synthase	39(55)
		Glov_3656	*sirA/cysG*	Siroheme synthase/ uroporphyrin-III C-methyltransferase	41(57)/40(57)
	Glov_2767	Glov_3657	*cbiO*	Cobalt ABC transporter, ATPase subunit	38(54) 35(56)
	Glov_2768	Glov_3658	*cbiQ*	Cobalt ABC transporter, inner membrane subunit	26(44) 23(45)
		Glov_3659	*cbiN*	Cobalt transport protein	42(57)
	Glov_2769	Glov_3660	*cbiM*	Cobalt transport protein	35(48) 54(68)
	Glov_0692	Glov_3718	*cobA*	Corrinoid:ATP adenosyltransferase	36(54) 46(67)
	Glov_3081		*cobU*	Bifunctional adenosylcobinamide kinase /adenosylcobinamide-phosphate guanylyltransferase	45(57)
	Glov_3082*	Glov_3678	*cobT*	Nicotinate-nucleotide-DMBA-phosphoribosyltransferase	38(57) 39(57)
	Glov_3083		*cobS*	Cobalamin synthase	35(51)
	Glov_3084		*cobC*	α-Ribazole-5'-phosphate phosphatase	25(43)
	Glov_3417		*cbiT*	Precorrin decarbocylase	33(54)
	Glov_3553		*cbiB*	Cobalamin biosynthesis protein	46(62)
	Glov_3554*	Glov_3679	*cobD/cbiP*	L-threonine O-3-phosphate decarboxylase/ cobyric acid synthase	34(51)/45(63)
					45(62)/35(51)

^#^ Chr. = chromosome.

* SZ chromosomal *cobT* and *cobD*/*cbiP* share 78% and 77% identity with their respective pSZ77 paralogs.

### Plasmid pSZ77

**Figure 3:**
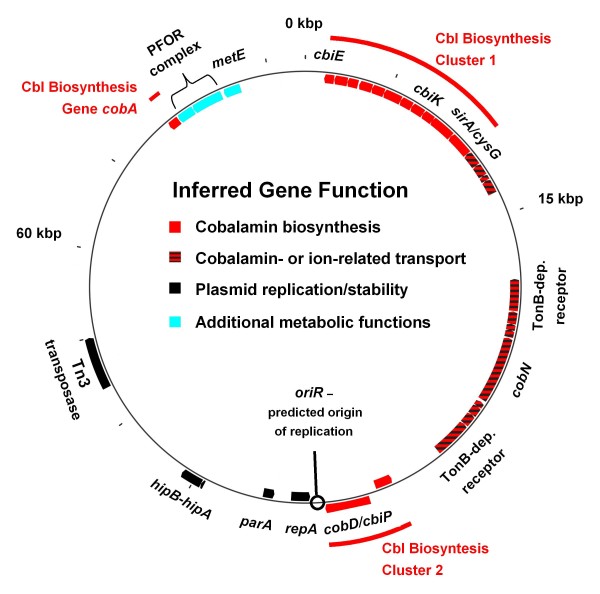
**Physical map of*G. lovleyi*strain SZ plasmid pSZ77.** Cobalamin (Cbl) biosynthesis clusters are shown in red. Transport-related genes such as the TonB-dependent receptors (Glov_3667 and Glov_3675) are red-black barred. Genes likely to play a role in pSZ77 replication or maintenance, such as *repA* (Glov_3681) are solid black. Additional metabolic functions, such as *metE* (cobalamin-independent methionine synthase) and PFOR complex genes (pyruvate ferredoxin/flavodoxin oxidoreductase A and B), are indicated in light blue. Genes lacking predicted function in *Geobacter* metabolism or pSZ77 replicon stability are not shown here, but are included in the pSZ77 map shown in Additional file 9.

The *G. lovleyi* genome sequence revealed the presence of a 77,113 bp circular plasmid, designated pSZ77. Out of the 81 total predicted plasmid-encoded ORFs, 32 were inferred to be involved in plasmid maintenance and stability along with cobalamin biosynthesis, membrane transport, or other functions likely to play critical roles to strain SZ’s metabolism (Figure 3). To date, *G. metallireducens* GS-15 and *P. propionicus* are the only closely related genomes to *G. lovleyi* that contain plasmids. The strain GS-15 plasmid is 14 kbp, and encodes genes predicted to be involved strictly in plasmid maintenance [38]. *P. propionicus* has a multipart genome comprised of a 4.0 Mbp chromosome and two plasmids. The larger 202 kbp plasmid pPRO1 has COGs class functions related to energy production, and encodes the two cytochrome *c* ammonia-forming nitrite reductases, NrfA and NrfH, and a *c-*type cytochrome of unspecified function. The smaller 31 kbp plasmid pPRO2 encodes two additional *c*-type cytochromes (Additional file 7). No *c*-type cytochromes are encoded on pSZ77, but 18% of the pSZ77 genes have predicted COGs class functions in coenzyme metabolism, mostly cobalamin biosynthesis (Table 2, Additional file 9).

### Plasmid maintenance

**Figure 4:**
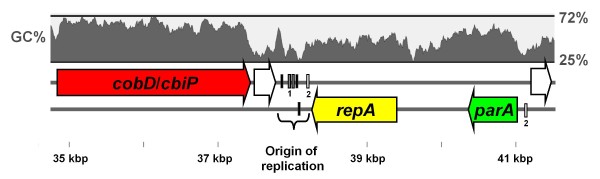
**Predicted replicon region of pSZ77.** The DnaA-binding sites found at a low-GC region 3' to the predicted *repA* ORF provide evidence that this region is the plasmid origin of replication (*oriR).* The repeats are not closely spaced, and do not resemble the tandem repeats found in the plasmids of *Geobacter metallireducens* and *Pelobacter propionicus*. Protein coding genes (arrows) are as follows: *repA*‒predicted replication initiation protein (yellow), *parA*‒predicted partitioning ATPase (green), *cobD/cbiP*: cobalamin synthesis pathway gene (red). The two unshaded genes are annotated as histone-binding proteins. Non-coding regions of interest (rectangles) are as follows: black = predicted DnaA protein-binding site, TT(C|A)TCCAC(A|G), grey = repeat 1, CGTGACAATTCATGC, white = repeat 2, TGTATAGACAATACAATA.

**Figure 5:**
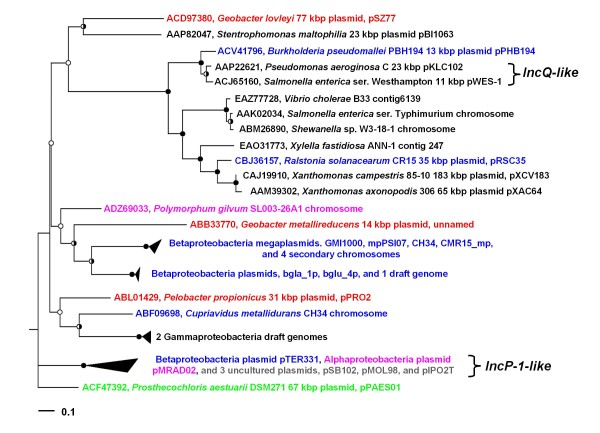
**Unrooted proML tree of 35 plasmid replication initiator protein (RepA) sequences aligning with pSZ77 RepA in PSI-BLAST.** The majority of RepA sequences in the tree are either from the *β-Proteobacteria* (blue, 15 in all) or the *γ-Proteobacteria* (black, 11 in all). Also represented are *α-Proteobacteria* (magenta) and *Chlorobi* (green). The SZ/KB-1 RepA (red) do not cluster with the other two *δ-Proteobacteria* RepA (red) suggesting a possible origin of the pSZ77 replicon region outside of the *δ-Proteobacteria*. Several of the RepA are chromosome-encoded, suggesting origins from mobilizable plasmids such as IncQ. The bootstrap values at each node with >90% support, 50–90% support and <50% support are indicated by black, half-shaded, and white circles, respectively. The scale bar indicates substitutions per site (0.1 = 1 in 10 amino acid residues).

Several pSZ77 protein-coding genes have putative function in plasmid replication or segregational stability and are associated with a predicted origin of replication, *oriR* (Figure 4). Glov_3681 encodes a protein with the replication initiation protein (RepA) domain (pfam04796). pSZ77 RepA may function in a complex with the SZ-chromosome-encoded DnaA protein to initiate plasmid replication [67,68] at predicted DnaA-binding sites [69] within the predicted *oriR* downstream of *repA* on pSZ77 (Figure 4). The pSZ77 RepA appears to share phylogenetic affiliation with the RepA of various plasmids from *β-* or *γ-Proteobacteria* (Figure 5), including an IncQ-like mobilizable plasmid [70] and an IncP-1-like environmental plasmid [71]. Yet, pSZ77 RepA does not share more than 39% aa identity (57% similarity) with the RepA encoded by either of these two characterized plasmids nor any other homologs in the public databases, including its two homologs from the plasmids of *δ-Proteobacteria* (identity 33–35%; similarity 49–55%). Upstream of pSZ77 *repA*, Glov_3684 encodes a ParA-type plasmid partitioning ATPase (Additional file 10) and may provide a mechanism for distribution of pSZ77 copies to daughter cells after division [72]. Glov_3687-Glov_3688 encode proteins homologous to HipB and HipA (Figure 4), a putative toxin-antidote system (Additional file 10), which may further contribute to pSZ77 stability [73]. Together, these observations suggest that the pSZ77 replicon belongs to a new plasmid incompatibility group with inferred mechanisms for both active partitioning and post-segregational stability.

### Plasmid gene clusters associated with cobalamin metabolism

The strain SZ chromosome contains several genes encoding cobalamin-dependent enzymes including ribonucleotide reductase, methionine synthase, and methylmalonyl-CoA mutase; genes that are found in all other *Geobacter* genomes (Additional file 11). In addition, the strain SZ chromosome encodes two PCE RDases that presumably require a cobalamin co-factor [44]. Accordingly, strain SZ has genes encoding all inferred functions necessary for *de novo* cobalamin biosynthesis plus a predicted outer membrane transport system, which may play a role in uptake of extracellular corrinoids. Unlike other *Geobacter* genomes, the majority of strain SZ cobalamin biosynthesis genes are encoded on the plasmid rather than the chromosome, localized in two gene clusters and a single gene locus (Table 4). The first cobalamin biosynthesis gene cluster comprises 15 consecutive or overlapping genes (Glov_3646 through Glov_3660) together encoding enzymes mediating the first 11 steps of *de novo* cobalamin biosynthesis and a cobalt ABC transporter. Genes 2 through 10 in the first cluster, *cbiJ-H-G-F-D-L-K-C-A-CysG*, along with one of the cobalt transport genes, *cbiN*, lack isofunctional homologs on the SZ chromosome suggesting the plasmid is essential when extracellular sources of cobalamin are lacking. The second cobalamin biosynthesis gene cluster comprises *cobT* (Glov_3678) on the reverse strand and *cobD*/*cbiP* (Glov_3679) on the leading strand, both showing evidence of duplication with genes on the SZ chromosome. CobT shares 78% aa identity with a chromosome-encoded CobT homolog. Similarly, the CobD/CbiP fusion shares 77% identity with a homologous fusion protein encoded on the SZ chromosome. The third locus is comprised of a single gene, *cobA* (Glov_3718), possibly involved in the adenosylation step of cobalamin biosynthesis. The pSZ77 CobA shares 36% aa identity with CobA encoded on the SZ chromosomal *cobA* locus. Genes encoding four of the final five steps of cobalamin biosynthesis, *cbiB*, and *cobUcobS*, and *cobC*, are found exclusively on the SZ chromosome (Table 4).

Nine genes highly similar to chromosomal orthologs are inferred in outer membrane transport and are situated between both cobalamin biosynthesis gene clusters on pSZ77 (Figure 3). Proteins encoded by genes at the 5’ and 3’ ends of this cluster, Glov_3667 and Glov_3675, were inferred by Pred-TMBB [27] to have up to 20 trans-membrane ß-strands characteristic of the TonB-dependent outer membrane receptors [74]. Proteins encoded by Glov_3670, Glov_3669, and Glov_3668 have sequence characteristics matching the ExbB, ExbD, and TonB proteins involved in outer membrane transport of iron siderophores or vitamin B_12_[74,75]. Glov_3671 encodes a protein annotated as a cobalt chelatase (CobN) (Figure 3) of the type involved in late cobalt insertion (i.e., “aerobic”) cobalamin biosynthesis [76]. The confirmed CobN cobalt chelatase of *Pseudomonas denitrificans* is associated with pCobS and pCobT subunits [77]. No genes predicted to encode homologs to these pCobS and pCobT accessory proteins were identified on the plasmid or chromosome of strain SZ, suggesting the pSZ77 CobN has a distinct function from its homolog in *P. denitrificans*. The pSZ77 putative CobN shared high aa similarity (79% identity) with a protein encoded by Gura_0774 on the *G. uraniireducens* chromosome, which is flanked by *tonB* receptor-related genes. These observations, coupled with its proximity to cobalamin biosynthesis clusters, suggest that the pSZ77-encoded CobN is associated with cobalamin metabolism in strain SZ, though its exact biochemical role is unknown. The *cobN-exbBexbDtonB* gene cluster matches chromosomal homologs in only two related genomes, *G. uraniireducens* and *P. propionicus*, and is predicted to function in the transport of iron siderophores, vitamin B_12_, or corrinoid precursors [74,75,78].

pSZ77 genes encoding cobalamin biosynthesis and cobalamin transport functions suggest that the plasmid might be essential for strain SZ growth. To examine the propensity for pSZ77 loss from *G. lovleyi* strain SZ, a series of protocols developed for curing non-essential plasmids from host organisms were assayed [19,20]. SDS at concentrations above 0.1% inhibited growth of strain SZ, but growth was stable over 17 consecutive transfers with ≤0.018% SDS. L-ascorbate at concentrations of up to 10 mM did not affect growth over at least nine consecutive transfers. Strain SZ grew with PCE or fumarate as electron acceptor over at least 40 consecutive transfers in cyanocobalamin-free and cyanocobalamin-amended medium. In samples from all treatments that allowed growth, PCR with the *repA*-targeted primer pair yielded amplicons of the expected size suggesting that the plasmid carrying the *repA* DNA fragment was maintained under the growth conditions tested. The observation that strain SZ grew in medium with or without cyanocobalamin amendment indicated that the cobalamin biosynthesis genes on pSZ77 are functional and that pSZ77 is maintained under growth conditions that do not require cobalamin biosynthesis. Our efforts to cure strain SZ of pSZ77 were unsuccessful due either to pSZ77 genes being essential to SZ metabolism or due to the effectiveness of plasmid maintenance/stability-related genes. For example, the pSZ77 *cobN*-associated TonB-type outer-membrane transport genes, which match chromosomal orthologs in *G. uraniireducens* and *P. propionicus*, may have been critical to strain SZ metabolism in the vitamin B_12_-amended cultures. The pSZ77 *tonBexbBexbD* genes associated with *cobN* do not correspond to a complete cluster on the SZ chromosome, suggesting that transport of cobalamin and/or other organometallic complexes made the plasmid essential under the cultivation conditions used. With these apparently essential functions, pSZ77 shares one of the defining traits of the significantly larger secondary replicons observed in *α-* and *β-Proteobacteria* termed “chromids” [79]. Alternatively, the pSZ77 genes predicted to contribute to plasmid stability, such as the partitioning ATPase (*parB*) or the toxin-antitoxin cluster (*hipB-hipA*) may be effective at maintaining the plasmid.

### Origins of pSZ77 genes and horizontal gene transfer

Sequence analyses of pSZ77 genes involved in plasmid maintenance or recombination, cobalamin biosynthesis, and amino acid biosynthesis suggested that a significant portion of the plasmid originated from outside the *Geobacteraceae*. The protein encoded by the putative *hipA* (Glov_3688) shares 75% aa identity with a homolog encoded on the large plasmid (pPRO1) of *P. propionicus*. The pSZ77 replication protein, RepA, shows only distant phylogenetic relatedness with its *Geobacter* and *Pelobacter* homologs (Figure 5) and the *repA* locus has an atypical codon usage (normalized CAI < 1.00, Additional file 12) for both the SZ chromosome and plasmid. The pSZ77 RepA protein is most similar to homologs of *γ-Proteobacteria* (Figure 5), such as the RepA encoded on plasmid pBI1063 (36% aa identity, 56% aa similarity) from *Stentrophomonas maltophilia*. The predicted plasmid-partitioning gene, *parA*, lacks *Geobacteraceae* or *Pelobacteraceae* homologs altogether (Additional file 10). Additionally, Glov_3721, encoding a predicted vitamin B_12_-independent methionine synthase (MetE), lacks homologs in the *Geobacteraceae* and is functionally redundant to the *metH* locus on the SZ chromosome (Additional file 11).

The pSZ77 cobalamin biosynthesis genes correspond to isofunctional homologs on the chromosomes of all *Geobacter* spp., but gene fusions and gene order within the cobalamin biosynthesis clusters on pSZ77 are uncharacteristic of cobalamin biosynthesis gene clusters in any other *Geobacter* genome. For instance, the gene fusions *sirA*/*cysG* in the first cobalamin biosynthesis cluster and *cobD*/*cbiP* in the second cluster fully align with their orthologs on the *Pelobacter carbinolicus* chromosome, but share similarity with unfused genes encoded at distinct ORFs in other *Geobacter* genomes. The proteins encoded by genes of the first pSZ77 cobalamin biosynthesis cluster share an average of 56% aa sequence identity with their orthologs on the *P. carbinolicus* chromosome, higher than the average pairwise identity (46%) between the proteomes of strain SZ and *P. carbinolicus*. However, most of the genes in the first and second cobalamin biosynthesis clusters have normalized CAIs > 1.00 (Additional file 12) suggesting the portion of pSZ77 encoding the cobalamin biosynthesis genes have resided in the SZ genome for sufficient time to ameliorate to its average codon usage [29,53].

### Distribution of SZ-like plasmids among dechlorinating *Geobacter* strains

A *G. lovleyi* strain sharing 99% 16S rRNA gene identity with strain SZ was isolated from the PCE-to-ethene-dechlorinating consortium KB-1 in order to examine its plasmid complement. This *Geobacter* isolate was found to be capable of dechlorinating PCE to *cis*-DCE, and was designated *G. lovleyi* strain KB-1. Metagenome sequencing of the KB-1 consortium and plasmid isolation and sequencing from the *Geobacter* strain KB-1 isolate revealed that strain KB-1 carries a 77,215 bp plasmid sharing high similarity with pSZ77 (99% identity). Although they were originally isolated from different locations and habitats (i.e., strain SZ from non-contaminated Su-Zi Creek freshwater sediment, South Korea [3], and strain KB-1 from a chlorinated solvent-contaminated aquifer in Ontario, Canada [5]), the PCE-to-*cis*-DCE dechlorinating *G. lovleyi* strain KB-1 and strain SZ share highly similar plasmids. The sequence differences between pSZ77 and the KB-1 assembly were comprised mainly of short insertions and deletions. An intact ORF encoding a recombinase on the KB-1 plasmid assembly corresponds to a pseudogene (Glov_3699) with a 119 bp deletion on pSZ77. A 136 bp insertion occurs on pSZ77 of strain SZ in an intergenic region upstream of Glov_3713 encoding a transposase. There are six apparent deletions in the KB-1 assembly relative to the pSZ77 DNA sequence, ranging in length from 1 bp to 136 bp (Additional file 13), but the majority of these deletions occur in positions in the assembly with two-fold sequence coverage or less, and so cannot be considered reliable. Out of the 79 intact ORFs shared between pSZ77 and the KB-1 assembly, only two single nucleotide polymorphisms could be found. Apparently, the pSZ77 and the KB-1 plasmid assemblies exhibit few sequence differences and little difference in gene repertoire. Likewise, the strain SZ genome shares ~99% identity with metagenome contigs obtained from consortium KB-1. Based on the geographically- and ecologically-disparate habitats of strain SZ and strain KB-1, the strictly anaerobic, non-spore-forming species *G. lovleyi* appears to be cosmopolitan.

The identification of the plasmid from strain KB-1 suggested that the 77 kbp plasmid is a shared feature of members of the organohalide-respiring *Geobacter* clade. To test this hypothesis, primers targeting the *repA* plasmid maintenance gene were used to amplify the pSZ77 *repA* gene from members of the organohalide-respiring *Geobacter* clade. Included in the analysis were four additional *G. lovleyi* isolates obtained from the chlorinated ethene-contaminated site, Fort Lewis, whose 16S rRNA gene sequences suggest a close phylogenetic relationship (Additional file 1) with strain SZ (99–100% nucleotide identity). Amplicons from the *repA*-targeted primers of the expected size and sequence were obtained with template DNA from strain SZ (positive control), strain KB-1 (not shown), and the BDI and KB-1 consortia (Additional file 14). No amplicons were obtained from any of the other organohalide-respiring *Geobacter* isolates, suggesting that the Fort Lewis soil *G. lovleyi* isolates Geo7.1, Geo7.2, Geo 7.3, and Geo7.4 do not contain a pSZ77-like plasmid. The primer set Geo564F/840R [21] was successfully used to amplify *Geobacteraceae* 16S rRNA genes from all samples. To further verify that the Fort Lewis *Geobacter* isolates lack plasmids, plasmid extractions were performed with biomass from the Fort Lewis *G. lovleyi* isolates, *G. thiogenes*, and *G. lovleyi* strain SZ. Only strain SZ DNA yielded the characteristic 77 kbp plasmid, and no bands indicative of a plasmid were observed in the other samples (data not shown). The lack of physical DNA in plasmid DNA extractions from *G. thiogenes* and the Fort Lewis *G. lovleyi* strains were consistent with the *repA*-targeted PCR results, suggesting neither *G. thiogenes* nor the Fort Lewis strains contain a similar plasmid. The lack of evidence for pSZ77-like plasmids in several PCE-respiring *Geobacter* suggested that pSZ77 does not play a direct or obligate role in organohalide respiration. Mechanisms for cobalamin biosynthesis or transport of corrinoids are essential for respiration with PCE and other chlorinated ethenes [44,80]. This implies that the *Geobacter* strains without a plasmid likely either have chromosome-encoded cobalamin biosynthesis genes or an efficient mechanism for uptake of extracellular corrinoids. While not indispensable to reductive dehalogenation across all *G. lovleyi* strains, pSZ77 may play an indirect adaptive role in PCE respiration in strains SZ and KB-1. For instance, pSZ77 may be important to strains SZ and KB-1 by allowing increased gene copy numbers or differential regulation of gene functions relevant for organohalide respiration [79]. The two cobalamin biosynthesis clusters on pSZ77 situated on either side of the TonB-encoding transport cluster (Figure 3) may thus provide strains SZ and KB-1 with a mechanism for simultaneous regulation of both cobalamin biosynthesis and scavenging for extracellular corrinoids.

## Conclusions

Phylogenetic analysis and sequence features related to respiratory metabolism support the affiliation of strain SZ within the *Geobacter* genus. Compared to other *Geobacter* genomes, strain SZ carries an unusually large quantity of laterally acquired DNA including the genomic island with duplicated *pceAB* gene clusters enabling strain SZ to respire PCE and TCE. The origin of the *pce*-genes is unclear given that they share low similarities with their top BlastP matches in *Desulfitobacterium* spp. The frequency and mechanism of transfer of the Pce genomic island are not yet understood, and as such the consequences of potential lateral transfer to non-dechlorinating *Geobacter* spp. are unclear; however, such events are obviously relevant for bioremediation. Up to 69% of strain SZ genes shared their top BlastP matches with other *Geobacter* spp. or *Pelobacter* spp. genomes (Table 1). At the same time, 9% of strain SZ genes did not have homologs in the sequenced representatives of the *δ-Proteobacteria* phylum. Notably, the SZ conjugative pilus *tra*-gene cluster comprises a portion of the largest chromosomal genomic island yet described in a *Geobacter* genome. The *tra*-genes may provide strain SZ with a mechanism for enhanced uptake of foreign DNA. Compared to other *Geobacter* genomes, strain SZ possesses fewer *c*-type cytochrome genes with no more than 12 heme binding motifs, which does not appear to limit the organism’s ability to respire oxidized metal species.

A distinguishing feature of the *G. lovleyi* genome is pSZ77, an extrachromosomal element carrying genes that are typically located on the chromosome in other *Geobacteraceae*. The phylogenetic origins of the pSZ77 *repA* and *parA* genes are unclear and may have been laterally acquired separately from the pSZ77 genes with chromosomal orthologs. Most notable among its genes with predominantly chromosomal homologs, pSZ77 encodes 15 out of the 24 genes required for *de novo* cobalamin biosynthesis. Although a nearly identical plasmid occurs in *G. lovleyi* strain KB-1, not all organohalide-respiring *Geobacterlovleyi* strains carry a non-chromosomal element, indicating that pSZ77 is not a marker for PCE reductive dechlorination within this species. On the other hand, unique genes and gene clusters, particularly the *pce*-genes, may provide discriminative detection of PCE-respiring *Geobacter* strains.

## Competing interests

The authors declare that they have no competing interests.

## Authors’ contributions

DDW, JKH, MRS, EPC, and KMR performed the experiments, and DDW carried out the computational analyses. LAH isolated strain KB-1 and assembled plasmid KB-1 from the metagenome data. EAE provided the metagenome sequence data. FEL conceived the study, and KTK participated in the design and coordination of the computational analyses. DDW prepared the figures and tables. DDW, LAH, KMR, KTK and FEL wrote the manuscript. All authors read and approved the final manuscript.

## Supplementary Material

Additional file 1 **Unrooted 16S rRNA gene tree showing organohalide-respiring δ-*Proteobacteria*from isolates or mixed cultures.** Selected related non-dechlorinating organisms (non-highlighted branches) are added to show divergence within genera. Click here for file

Additional file 2 **Codon usage of *pce*-genes and adjacent genes encoded on the Pce genomic island in comparison to all strain SZ chromosomal genes using the codon adaptation index (CAI).** Normalized CAI < 1.00 (red font) indicates a possible laterally acquired gene and is scored below genomic expected CAI at a 5% level of significance (genomes.urv.es/CAIcal/E-CAI). Click here for file

Additional file 3 **Circular genome map of the*G. lovleyi*strain SZ chromosome (CP001089).** From outside to center: COG categories of genes on forward strand, COG categories of genes on reverse strand, percent GC content, GC skew, and percent blastx identity of strain SZ ORFs to ORFs on the chromosomes of *Pelobacter propionicus* (CP000482), *G. uraniireducens*, and *G. sulfurreducens*. Genomic island regions, six in all, are indicated by pink arcs and are characterized by genes lacking blast matches in a majority of other *Geobacter*/*Pelobacter* spp. genomes (e.g. the three innermost circles), suggesting horizontal gene transfer. The genomic island Pce harbors the *pce*-gene cluster, encoding the PCE reductive dehalogenases, and exhibits nucleotide sequence deviations in GC% and GC-skew relative to the entire strain SZ chromosome. The chromosome map was generated using GenomeViz [81]. Click here for file

Additional file 4 **Codon usage of predicted F-factor conjugative pilus*tra*-genes in comparison to all strain SZ chromosomal genes using the codon adaptation index (CAI).** A majority of genes in the *tra*-pilus cluster have normalized CAI < 1.00 (red font), but are interspersed with genes having normalized CAI > 1.00, indicating sufficient residence time in strain SZ for the cluster to partially ameliorate to chromosomal codon usage. Click here for file

Additional file 5 **Inferred*c*-type cytochrome genes on the*G. lovleyi*strain SZ chromosome.** Click here for file

Additional file 6 **Inferred c-type cytochrome genes in*Pelobacter propionicus*DSM 2379.** Click here for file

Additional file 7 **Electron-transfer proteins encoded on the*G. lovleyi*strain SZ chromosome.** Click here for file

Additional file 8 **Inferred reactive-oxygen-species-responsive or oxygen-reducing genes in*G. lovleyi*strain SZ.** Click here for file

Additional file 9 **Circular genome map of the*G. lovleyi*strain SZ plasmid pSZ77 (CP001090).** From outside to center: COG categories of genes on forward strand, COG categories of genes on reverse strand, percent GC content, GC skew, percent blastx identity of SZ ORFs to plasmids of *Pelobacter propionicus* (CP000483 and CP000484), and percent blastx identity to the chromosomes of *Pelobacter propionicus* (CP000482), *Geobacter uraniireducens*, and *G. sulfurreducens*. The plasmid map was generated using Genome Viz [81]. Click here for file

Additional file 10 pSZ77 genes related to plasmid replication, maintenance, and recombination. Click here for file

Additional file 11 **Inferred cobalamin-dependent genes on the*G. lovleyi*strain SZ chromosome.** Click here for file

Additional file 12 **Codon usage of pSZ77 genes in comparison to strain SZ chromosomal genes using the codon adaptation index (CAI).** Out of 79 total predicted plasmid genes, 42 have normalized CAI > 1.00, indicating that over half of pSZ77 genes have resided in the strain SZ genome for sufficient time to ameliorate their codon usage to the chromosome. The predicted replication/maintenance genes *repA* and *parA* have normalized CAI < 1.00 (red font) [82]. Click here for file

Additional file 13 DNA sequence differences between the pSZ77 and the KB-1 plasmid assemblies. Click here for file

Additional file 14 **Detection of the pSZ77*repA*gene and*Geobacteraceae*spp.** 16S rRNA genes in pure and mixed cultures containing *Geobacter* strains with specific PCR primers. Top row: *repA* gene-targeted PCR (565 bp amplicon) and bottom row *Geobacteraceae* 16S rRNA gene-targeted PCR (312 bp amplicon). The arrows indicate the expected PCR amplicons. Lane 1: 1 kb Plus DNA ladder (Invitrogen), Lane 2: no template (negative control), Lane 3: *G. lovleyi* strain SZ (positive control), Lanes 4 and 5: consortium BDI, Lane 6: consortium KB-1, Lane 7: Ft. Lewis isolate 7.1, Lane 8: Ft. Lewis isolate 7.2, Lane 9: Ft. Lewis isolate 7.3, Lane 10: Ft. Lewis isolate 7.4, Lane 11: *G. thiogenes*. Click here for file

## References

[B1] Coates JD Phillips EJP Lonergan DJ Jenter H Lovley DR Isolation of Geobacter species from diverse sedimentary environments Appl Environ Microbiol 1996 62 1531 1536 8633852 10.1128/aem.62.5.1531-1536.1996 PMC167928

[B2] Anderson RT Vrionis HA Ortiz-Bernad I Resch CT Long PE Dayvaul R Karp K Marutzky S Metzler DR Peacock A Stimulating the in situ activity of Geobacter species to remove uranium from the groundwater of a uranium-contaminated aquifer Appl Environ Microbiol 2003 69 5884 5891 10.1128/AEM.69.10.5884-5891.2003 14532040 PMC201226

[B3] Sung Y Fletcher KE Ritalahti KM Apkarian RP Ramos-Hernández N Sanford RA Mesba NM Löffler FE Geobacter lovleyi sp. nov. strain SZ, a novel metal-reducing and tetrachloroethene-dechlorinating bacterium Appl Environ Microbiol 2006 69 2775 2782 16597982 10.1128/AEM.72.4.2775-2782.2006 PMC1448980

[B4] DeWever H Cole JR Fettig MR Hogan DA Tiedje JM Reductive dehalogenation of trichloroacetic acid by Trichlorobacter thiogenes gen. nov., sp. nov Appl Environ Microbiol 2000 66 2297 2301 10.1128/AEM.66.6.2297-2301.2000 10831402 PMC110515

[B5] Duhamel M Edwards EA Microbial composition of chlorinated ethene-degrading cultures dominated by Dehalococcoides FEMS Microbiol Ecol 2006 58 538 549 10.1111/j.1574-6941.2006.00191.x 17117995

[B6] Amos BK Sung Y Fletcher KE Gentry TJ Wu W-M Criddle CS Zhou J Löffer FE Detection and quantification of Geobacter lovleyi strain SZ: Implications for bioremediation at tetrachloroethene- and uranium-impacted sites Appl Environ Microbiol 2007 73 6898 6904 10.1128/AEM.01218-07 17827319 PMC2074934

[B7] Costanza J Fletcher KE Löffler FE Pennell KD Fate of TCE in heated Fort Lewis soil Environ Sci Technol 2009 43 909 914 10.1021/es802508x 19245035

[B8] Amos BK Suchomel EJ Pennell KD Löffler FE Spatial and temporal distributions of Geobacter lovleyi and Dehalococcoides spp. during bioenhanced PCE-NAPL dissolution Environ Sci Technol 2009 43 1977 1985 10.1021/es8027692 19368201

[B9] Strycharz SM Woodard TL Johnson JP Nevin KP Sanford RA Löffler FE Lovley DR Graphite electrode as a sole electron donor for reductive dechlorination of tetrachloroethene by Geobacter lovleyi Appl Environ Microbiol 2008 2008 5943 5947 18658278 10.1128/AEM.00961-08 PMC2565976

[B10] Sung Y Ritalahti KM Sanford RA Urbance JW Flynn SJ Tiedje JM Loffler FE Characterization of two tetrachloroethene-reducing, acetate-oxidizing anaerobic bacteria and their description as Desulfuromonas michiganensis sp. nov Appl Environ Microbiol 2003 69 2964 2974 10.1128/AEM.69.5.2964-2974.2003 12732573 PMC154526

[B11] Sanford RA Cole JR Tiedje JM Characterization and description of Anaeromyxobacter dehalogenans gen. nov., sp. nov., an aryl-halorespiring facultative anaerobic myxobacterium Appl Environ Microbiol 2002 68 893 900 10.1128/AEM.68.2.893-900.2002 11823233 PMC126698

[B12] Thomas SH Sanford RA Amos BK Leigh MB Cardenas E Löffler FE Unique ecophysiology among U(VI)-reducing bacteria as revealed by evaluation of oxygen metabolism in Anaeromyxobacter dehalogenans strain 2CP-C Appl Environ Microbiol 2010 76 176 183 10.1128/AEM.01854-09 19897758 PMC2798628

[B13] Thomas SH Wagner RD Arakaki AK Skolnick J Kirby JR Shimkets LJ Sanford RA Löffler FE The mosaic genome of Anaeromyxobacter dehalogenans strain 2CP-C suggests an aerobic common ancestor to the delta-proteobacteria PLoS One 2008 3 e2103 10.1371/journal.pone.0002103 18461135 PMC2330069

[B14] Fletcher KE Costanza J Pennell KP Löffler FE Electron donor availability for microbial reductive processes following thermal treatment Water Res 2011 45 6625 6636 10.1016/j.watres.2011.09.033 22048015

[B15] Löffler FE Sanford RA Ritalahti KM Enrichment, cultivation, and detection of reductively dechlorinating bacteria Methods Enzymol 2005 397 77 111 16260286 10.1016/S0076-6879(05)97005-5

[B16] Löffler FE Champine JE Ritalahti KM Sprague SJ Tiedje JM Complete reductive dechlorination of 1,2-dichloropropane by anaerobic bacteria Appl Environ Microbiol 1997 63 2870 2875 16535654 10.1128/aem.63.7.2870-2875.1997 PMC1389209

[B17] Amos BK Christ JA Abriola LM Pennell KD Löffler FE Experimental evaluation and mathematical modeling of microbially enhanced tetrachloroethene (PCE) dissolution Environ Sci Technol 2007 43 1977 1985 17328210 10.1021/es061438n

[B18] Duhamel M Mo K Edwards EA Characterization of a highly enriched Dehalococcoides-containing culture that grows on vinyl chloride and trichloroethene Appl Environ Microbiol 2004 70 5538 5545 10.1128/AEM.70.9.5538-5545.2004 15345442 PMC520850

[B19] El-Mansi M Anderson KJ Inche CA Knowles LK Platt DJ Isolation and curing of the Klebsiella pneumonia large indigenous plasmid using sodium dodecyl sulphate Res Microbiol 2000 151 201 208 10.1016/S0923-2508(00)00140-6 10865947

[B20] Ramesh A Halami PM Chandrashekar A Ascorbic acid-induced loss of a pediocin-encoding plasmid in Pediococcus acidilactici CFR K7 World J Microbiol Biotechnol 2001 16 695 697

[B21] Cummings DE Snoeyenbos-West OL Newby DT Niggemyer AM Lovley DR Achenbach LA Rosenzweig RF Diversity of Geobacteraceae species inhabiting metal-polluted freshwater lake sediments ascertained by 16S rDNA analyses Microb Ecol 2003 46 257 269 14708750 10.1007/s00248-005-8002-3

[B22] Kieser T Factors affecting the isolation of cccDNA from Streptococcus lividans and Escherichia coli Plasmid 1984 12 19 36 10.1016/0147-619X(84)90063-5 6387733

[B23] Sobecky PA Mincer TJ Chang MC Helinski DR Plasmids isolated from marine sediment microbial communities contain replication and incompatibility regions unrelated to those of known plasmid groups Appl Environ Microbiol 1997 63 888 895 9055407 10.1128/aem.63.3.888-895.1997 PMC168381

[B24] Sambrook J Russell D Molecular cloning: A laboratory manual, 3rd edn: Cold Spring Harbor Laboratory 2001

[B25] Wilson K Preparation of genomic DNA from bacteria Mol Biol 2001 2 4 10.1002/0471142727.mb0204s56 18265184

[B26] Altschul SF Madden TL Schaffer AA Zhang J Zhang Z Miller W Lipman DJ Gapped BLAST and PSI-BLAST: a new generation of protein database search programs Nucleic Acids Res 1997 25 3389 3402 10.1093/nar/25.17.3389 9254694 PMC146917

[B27] Bagos PG Liakopoulos TD Spyropoulos IC Hamodrakas SJ A Hidden Markov Model method, capable of predicting and discriminating ß-barrel outer membrane proteins BMC Bioinforma 2004 5 29 10.1186/1471-2105-5-29 PMC385222 15070403

[B28] Kurtz S Choudhuri JV Ohlebusch E Schleiermacher C Stoye J Giegerich R REPuter: The manifold applications of repeat analysis on a genomic scale Nucleic Acids Res 2001 29 4633 4642 10.1093/nar/29.22.4633 11713313 PMC92531

[B29] Puigbò P Bravo IG Garcia S E-CAI: a novel server to estimate an expected value of Codon Adaptation Index (eCAI) BMC Bioinforma 2008 9 65 10.1186/1471-2105-9-65 PMC2246156 18230160

[B30] Gao F Zhang C-T Ori-Finder: A web-based system for finding oriCs in unannotated bacterial genomes BMC Bioinforma 2008 9 79 10.1186/1471-2105-9-79 PMC2275245 18237442

[B31] Edgar RC MUSCLE: a multiple sequence alignment method with reduced time and space complexity BMC Bioinforma 2004 5 113 10.1186/1471-2105-5-113 PMC517706 15318951

[B32] Felsenstien J PHYLIP—Phylogeny Inference Package (version 3.2) Cladistics 1989 5 164 166

[B33] Cole JR Wang Q Cardenas E Fish J Chai B Farris RJ Kulam-Syed-Mohideen AS McGarrell DM Marsh T Garrity GM The Ribosomal Database Project: improved alignments and new tools for rRNA analysis Nucleic Acids Res 2009 37 D141 D145 10.1093/nar/gkn879 19004872 PMC2686447

[B34] Letunic I Bork P Interactive Tree Of Life (iTOL): an online tool for phylogenetic tree display and annotation Bioinformatics 2007 23 127 128 10.1093/bioinformatics/btl529 17050570

[B35] Altshul SF Gish W Miller W Myers EW Lipman DJ Basic local alignment search tool J Mol Biol 1990 215 403 410 2231712 10.1016/S0022-2836(05)80360-2

[B36] Stothard P Wishart DS Circular genome visualization and exploration using CGview Bioinformatics 2005 21 537 539 10.1093/bioinformatics/bti054 15479716

[B37] Methé BA Nelson KE Eisen JA Paulsen IT Nelson W Heidelberg JF Wu D Wu M Ward N Beanan MJ Genome of Geobacter sulfurreducens: Metal reduction in subsurface environments Science 2003 302 1967 1969 10.1126/science.1088727 14671304

[B38] Aklujar M Krushkal J DiBartolo G Lapidus A Land ML Lovley DR The genome sequence of Geobacter metallireducens: features of metabolism, physiology, and regulation common and dissimilar to Geobacter sulfurreducens BMC Microbiol 2009 9 109 10.1186/1471-2180-9-109 19473543 PMC2700814

[B39] Aklujkar M Young ND Holmes D Chavan M Risso C Kiss HE Han CS Land ML Lovley DR The genome of Geobacter bemidjiensis, exemplar for the subsurface clade of Geobacter species that predominate in Fe(III)-reducing subsurface environments BMC Genomics 2010 11 490 10.1186/1471-2164-11-490 20828392 PMC2996986

[B40] Shelobolina ES Vrionis HA Findlay RH Lovley DR Geobacter uraniireducens sp. nov., isolated from subsurface sediment undergoing uranium bioremediation Int J Syst Evol Microbiol 2008 58 1075 1078 10.1099/ijs.0.65377-0 18450691

[B41] Prakash O Thomas GM Dalton DD Chin K-J Green SJ Akob DM Wanger G Kostka JE Geobacter daltonii sp. nov., an Fe(III)- and uranium(VI)-reducing bacterium isolated from a shallow subsurface exposed to mixed heavy metal and hydrocarbon contamination Int J Syst Evol Microbiol 2010 60 546 553 10.1099/ijs.0.010843-0 19654355

[B42] Maillard J Regeard C Holliger C Isolation and characterization of Tn-Dha1, a transposon containing the tetrachloroethene reductive dehalogenase of Desulfitobacterium hafniense strain TCE1 Environ Microbiol 2005 7 107 117 10.1111/j.1462-2920.2004.00671.x 15643941

[B43] Furukawa K Suyama A Tsuboi Y Futagami T Goto M Biochemical and molecular characterization of a tetrachloroethene dechlorinating Desulfitobacterium sp. strain Y51: a review J Ind Microbiol Technol 2005 32 534 541 10.1007/s10295-005-0252-z 15959725

[B44] Maillard J Schumacher W Vazquez F Regeard C Hagen WR Holliger C Characterization of the corrinoid iron-sulfur protein tetrachloroethene reductive dehalogenase of Dehalobacter restrictus Appl Environ Microbiol 2003 69 4628 4638 10.1128/AEM.69.8.4628-4638.2003 12902251 PMC169082

[B45] Futagami T Yamaguchi T Nakayama S-i Goto M Furukawa K Effects of chloromethanes on growth of and deletion of the pce gene cluster in dehalorespiring Desulfitobacterium hafniense Strain Y51 Appl Environ Microbiol 2006 72 5998 6003 10.1128/AEM.00979-06 16957221 PMC1563609

[B46] Morita Y Futagami T Goto M Furukawa K Functional characterization of the trigger factor protein PceT of tetrachloroethene-dechlorinating Desulfitobacterium hafniense Y51 Appl Microbiol Biotechnol 2009 83 775 781 10.1007/s00253-009-1958-z 19347335

[B47] McMurdie PJ Behrens SF Holmes S Spormann AM Unusual codon bias in vinyl chloride reductase genes of Dehalococcoides species Appl Environ Microbiol 2007 73 2744 2747 10.1128/AEM.02768-06 17308190 PMC1855607

[B48] McMurdie PJ Hug L Edwards EA Holmes S Spormann AM Site-specific mobilization of vinyl chloride respiration islands by a mechanism common in Dehalococcoides BMC Genomics 2011 12 287 10.1186/1471-2164-12-287 21635780 PMC3146451

[B49] McMurdie PJ Behrens S Müller JA Göke J Ritalahti KM Wagner R Goltsman E Lapidus A Holmes S Löffler FE Localized plasticity in the streamlined genomes of vinyl chloride respiring Dehalococcoides PLoS Genetics 2009 5 e1000714 10.1371/journal.pgen.1000714 19893622 PMC2764846

[B50] Merkl R A comparative categorization of protein function encoded in Bacterial or Archaeal genomic islands J Mol Evol 2006 62 1 14 10.1007/s00239-004-0311-5 16341468

[B51] Lawley TD Klimke WA Gubbins MJ Frost LS F factor conjugation is a true type IV secretion system FEMS Microbiol Lett 2003 224 1 15 10.1016/S0378-1097(03)00430-0 12855161

[B52] Lawley TD Gilmour MW Gunton JE Tracz DM Taylor DE Functional and mutational analysis of conjugative transfer region 2 (Tra2) from the IncHI1 Plasmid R27 J Bacteriol 2003 185 581 591 10.1128/JB.185.2.581-591.2003 12511505 PMC145343

[B53] Lawrence JG Ochman H Molecular archaeology of the Escherichia coli genome Proc Natl Acad Sci U S A 1998 95 9413 9417 10.1073/pnas.95.16.9413 9689094 PMC21352

[B54] Butler JE Young ND Lovley DR Evolution of electron transfer out of the cell: comparative genomics of six Geobacter genomes BMC Genomics 2010 11 40 10.1186/1471-2164-11-40 20078895 PMC2825233

[B55] El-Naggar MY Wanger G Leung KM Yuzvinsky TD Southam G Yang J Lau WM Nealson KH Gorby YA Electrical transport along bacterial nanowires from Shewanella oneidensis MR-1 Proc Natl Acad Sci U S A 2010 107 18127 18131 10.1073/pnas.1004880107 20937892 PMC2964190

[B56] Inoue K Leang C Franks AE Woodward TL Nevin KP Lovley DR Specific localization of the c-type cytochrome OmcZ at the anode surface in current-producing biofilms of Geobacter sulfurreducens Environ Microbiol Rep 2011 3 211 217 10.1111/j.1758-2229.2010.00210.x 23761253

[B57] Mehta T Coppi MV Childers SE Lovley DR Outer membrane c-type cytochromes required for Fe(III) and Mn(IV) oxide reduction in Geobacter sulfurreducens Environ Microbiol 2005 71 8634 8641 10.1128/AEM.71.12.8634-8641.2005 PMC1317342 16332857

[B58] Reguera G McCarthy KD Mehta T Nicoll JS Tuominen MT Lovley DR Extracellular electron transfer via microbial nanowires Nature 2005 435 1098 1101 10.1038/nature03661 15973408

[B59] Cologgi DL Lampa-Pastrick S Speers AM Kelly SD Reguera G Extracellular reduction of uranium viaGeobacterconductive pili as a protective cellular mechanism. Proceedings of the National Academy of Sciences of the United States of America 2011 www.pnas.org/cgi/doi/10.1073/pnas.1108616108 10.1073/pnas.1108616108 PMC3174638 21896750

[B60] Butler JE Young ND Lovley DR Evolution from a respiratory ancestor to fill syntrophic and fermentative niches: Comparative genomics of six Geobacteraceae species BMC Genomics 2009 10 103 10.1186/1471-2164-10-103 19284579 PMC2669807

[B61] Marietou A Richardson D Cole J Mohan S Nitrate reduction by Desulfovibrio desulfuricans: a periplasmic nitrate reductase system that lacks NapB, but includes a unique tetraheme c-type cytochrome, NapM FEMS Microbiol Lett 2005 248 217 225 10.1016/j.femsle.2005.05.042 15972253

[B62] Mahadevan R Bond DR Butler JE Esteve-Nuñez A Coppi MV Palsson BO Schilling CH Lovley DR Characterization of metabolism in the Fe(III)-reducing organism Geobacter sulfurreducens by constraint-based modeling Appl Environ Microbiol 2006 72 1558 1568 10.1128/AEM.72.2.1558-1568.2006 16461711 PMC1392927

[B63] Nuñez C Esteve-Nuñez A Giometti C Tollaksen S Khare T Lin W Lovley DR Methé BA DNA microarray and proteomic analysis of the RpoS regulon in Geobacter sulfurreducens J Bacteriol 2006 188 2792 2800 10.1128/JB.188.8.2792-2800.2006 16585740 PMC1446979

[B64] Das A Silaghi-Dumitrescu R Ljungdahl LG Kurtz DM Cytochrome bd oxidase, oxidative stress, and dioxygen tolerance of the strictly anaerobic bacterium Moorella thermoacetica J Bacteriol 2005 187 2297 2301 10.1128/JB.187.7.2297-2307.2005 15743950 PMC1064043

[B65] Mouser PJ Holmes DE Perpetua LA DiDonato R Postier B Liu A Lovley DR Quantifying expression of Geobacter spp. oxidative stress genes in pure culture and during in situ uranium bioremediation ISME J 2009 3 454 465 10.1038/ismej.2008.126 19129865

[B66] Lovley DR Extracellular electron transfer: wires, capacitors, iron lungs, and more Geobiology 2008 6 225 231 10.1111/j.1472-4669.2008.00148.x 18393985

[B67] Sharma S Sathyanarayan BK Bird JG Hoskins JR Lee B Wickner S Plasmid P1 RepA is homologous to the F plasmid RepE class of initators J Biol Chem 2003 279 6027 6034 10.1074/jbc.M310917200 14634015

[B68] Gd S Giraldo R Ruiz-Echevarría MJ Espinosa M Díaz-Orejas R Replication and control of circular bacterial plasmids Microbiol Mol Biol Rev 1998 62 434 464 9618448 10.1128/mmbr.62.2.434-464.1998 PMC98921

[B69] Schaper S Messer W Interaction of the initiator protein DnaA of Escherichia coli with its DNA target J Biol Chem 1995 270 17622 17626 10.1074/jbc.270.29.17622 7615570

[B70] Doublet B Granier SA Robin F Bonnet R Fabre L Brisabois A Cloeckaert A Weill F-X Novel plasmid-encoded ceftazidime-hydrolyzing CTX-M-53 extended-spectrum β-lactamase from Salmonella enteric serotypes Westhampton and Senftenberg Antimicrob Agents Chemother 2009 53 1944 1951 10.1128/AAC.01581-08 19273683 PMC2681554

[B71] Mela F Fritsche K Boersma H Elsas JDv Bartels D Meyer F Boer Wd Veen JAv Leveau JHJ Comparative genomics of the pIPO2/pSB102 family of environmental plasmids: sequence, evolution, and ecology of pTer331 isolated from Collimonas fungivorans Ter331 FEMS Microbiol Ecol 2008 66 45 62 10.1111/j.1574-6941.2008.00472.x 18355297

[B72] Hayes F The partition system of multidrug resistance plasmid TP228 includes a novel protein that epitomized an evolutionarily distinct subgroup of the ParA superfamily Mol Microbiol 2000 37 528 541 10931346 10.1046/j.1365-2958.2000.02030.x

[B73] Nordström K Austin SJ Mechanisms that contribute to the stable segregation of plasmids Annu Rev Genet 1989 23 37 69 10.1146/annurev.ge.23.120189.000345 2694936

[B74] Ferguson AD Deisenhofer J TonB-dependent receptors–structural perspectives Biochim Biophys Acta 2002 1565 318 332 10.1016/S0005-2736(02)00578-3 12409204

[B75] Higgs PI Myers PS Postle K Interactions in the TonB-dependent energy transduction complex: ExbB and ExbD form homomultimers J Bacteriol 1998 180 6031 6038 9811664 10.1128/jb.180.22.6031-6038.1998 PMC107680

[B76] Scott AI Roessner CA Biosynthesis of cobalamin (vitamin B12) Biochem Soc Trans 2002 30 613 620 12196148 10.1042/bst0300613

[B77] Debussche L Couder M Thibaut D Cameron B Crouzet J Blanche F Assay, purification, and characterization of cobaltochelatase, a unique complex enzyme catalyzing cobalt insertion in hydrogenobyrinic acid a, c-diamide during coenzyme B12 biosynthesis in Pseudomonas denitrificans J Bacteriol 1992 174 7445 7451 1429466 10.1128/jb.174.22.7445-7451.1992 PMC207441

[B78] Braun V Energy-coupled transport and signal transduction through the Gram-negative outer membrane via TonB-ExbB-ExbD-dependent receptor proteins FEMS Microbiol Rev 1995 16 295 307 10.1111/j.1574-6976.1995.tb00177.x 7654405

[B79] Harrison PW Lower RPJ Kim NKD Young JPW Introducing the bacterial 'chromid' not a chromosome, not a plasmid Trends Microbiol 2010 18 141 148 10.1016/j.tim.2009.12.010 20080407

[B80] He J Holmes VF Lee PKH Alvarez-Cohen L Influence of Vitamin B12 and cocultures on the growth of Dehalococcoides isolates in defined medium Appl Environ Microbiol 2007 73 2847 2853 10.1128/AEM.02574-06 17337553 PMC1892872

[B81] Ghai R Hain T Chakraborty T GenomeViz: visualizing microbial genomes BMC Bioinformatics 2004 5 198 203 10.1186/1471-2105-5-198 15601465 PMC544189

[B82] Slater JH Weightman AJ Hall BG Dehalogenase genes of Pseudomonas putida PP3 on chromosomally located transposable elements Mol Biol Evol 1985 2 557 567 2835577 10.1093/oxfordjournals.molbev.a040366

