# Supplementary material for: Genomic determinants of organohalide-respiration in Geobacter lovleyi, an unusual member of the Geobacteraceae
Source: BMC Genomics. 2012 May 22;13:200. doi: 10.1186/1471-2164-13-200 (PMC3403914; doi:10.1186/1471-2164-13-200)
Supplement: Additional file 1 — Unrooted 16S rRNA gene tree showing organohalide-respiring δ-Proteobacteriafrom isolates or mixed cultures. Selected related non-dechlorinating organisms (non-highlighted branches) are added to show divergence within genera. [file 1471-2164-13-200-S1.doc]

**
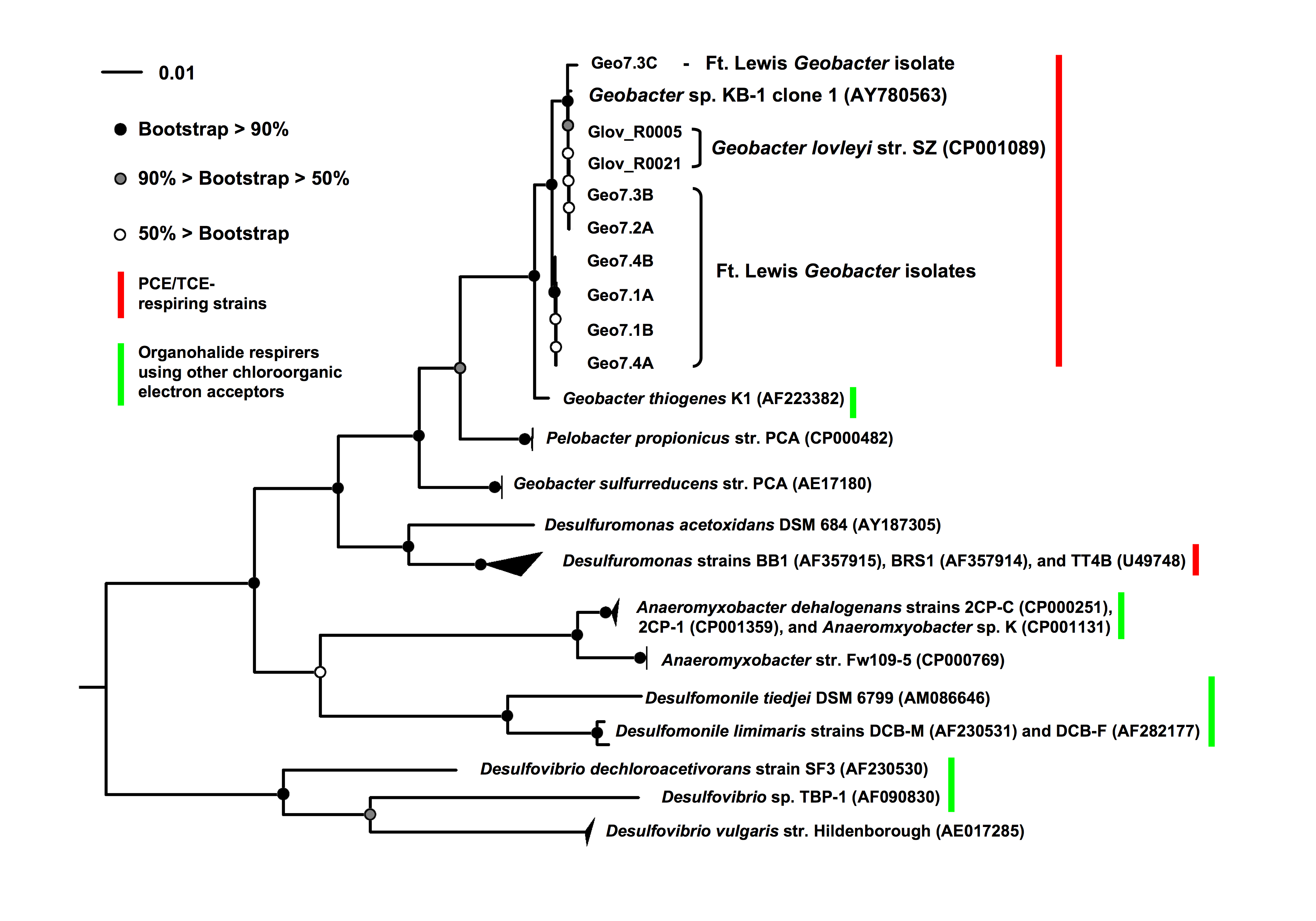
**

**Additional file 1:** Unrooted 16S rRNA gene tree showing organohalide-respiring -*Proteobacteria* from isolates or mixed cultures. Selected related non-dechlorinating organisms (non-highlighted branches) are added to show divergence within genera.
