# Supplementary material for: Genomic determinants of organohalide-respiration in Geobacter lovleyi, an unusual member of the Geobacteraceae
Source: BMC Genomics. 2012 May 22;13:200. doi: 10.1186/1471-2164-13-200 (PMC3403914; doi:10.1186/1471-2164-13-200)
Supplement: Additional file 2 — Codon usage of pce-genes and adjacent genes encoded on the Pce genomic island in comparison to all strain SZ chromosomal genes using the codon adaptation index (CAI). Normalized CAI < 1.00 (red font) indicates a possible laterally acquired gene and is scored below genomic expected CAI at a 5% level of significance (genomes.urv.es/CAIcal/E-CAI). [file 1471-2164-13-200-S2.doc]

**Additional file 2:** Codon usage of *pce*-genes and adjacent genes encoded on the Pce genomic island in comparison to all strain SZ chromosomal genes using the codon adaptation index (CAI). Normalized CAI < 1.00 (red font) indicates a possible laterally acquired gene and is scored below genomic expected CAI at a 5% level of significance (genomes.urv.es/CAIcal/E-CAI).

| SZ chromosome locus | Function | Length (bp) | Normalized CAI* |
| --- | --- | --- | --- |
| Glov_2865 | IstB domain ATP-binding protein | 744 | 1.03 |
| Glov_2866 | Integrase catalytic region | 1512 | 1.11 |
| Glov_2868 | PceT - Peptidylprolyl isomerase | 936 | 0.92 |
| Glov_2869 | PceC – FMN-binding domain protein | 1149 | 0.85 |
| Glov_2870 | PceA – reductive dehalogenase, catalytic subunit | 1545 | 0.87 |
| Glov_2871 | PceB – reductive dehalogenase membrane anchor subunit | 330 | 0.85 |
| Glov_2872 | PceA – reductive dehalogenase, catalytic subunit | 1545 | 0.87 |
| Glov_2873 | PceB – reductive dehalogenase membrane anchor subunit | 330 | 0.85 |
| Glov_2874 | Aldehyde dehyrogenase | 1416 | 0.95 |
| Glov_2875 | Transposase IS204/IS1001/IS1096/IS1165 | 1176 | 0.96 |
| * Normalized to codon usage over the entire strain SZ chromosome | | | |
