# Supplementary material for: Genomic determinants of organohalide-respiration in Geobacter lovleyi, an unusual member of the Geobacteraceae
Source: BMC Genomics. 2012 May 22;13:200. doi: 10.1186/1471-2164-13-200 (PMC3403914; doi:10.1186/1471-2164-13-200)
Supplement: Additional file 3 — Circular genome map of theG. lovleyistrain SZ chromosome (CP001089). From outside to center: COG categories of genes on forward strand, COG categories of genes on reverse strand, percent GC content, GC skew, and percent blastx identity of strain SZ ORFs to ORFs on the chromosomes of Pelobacter propionicus (CP000482), G. uraniireducens, and G. sulfurreducens. Genomic island regions, six in all, are indicated by pink arcs and are characterized by genes lacking blast matches in a majority of other Geobacter/Pelobacter spp. genomes (e.g. the three innermost circles), suggesting horizontal gene transfer. The genomic island Pce harbors the pce-gene cluster, encoding the PCE reductive dehalogenases, and exhibits nucleotide sequence deviations in GC% and GC-skew relative to the entire strain SZ chromosome. The chromosome map was generated using GenomeViz [81]. [file 1471-2164-13-200-S3.doc]

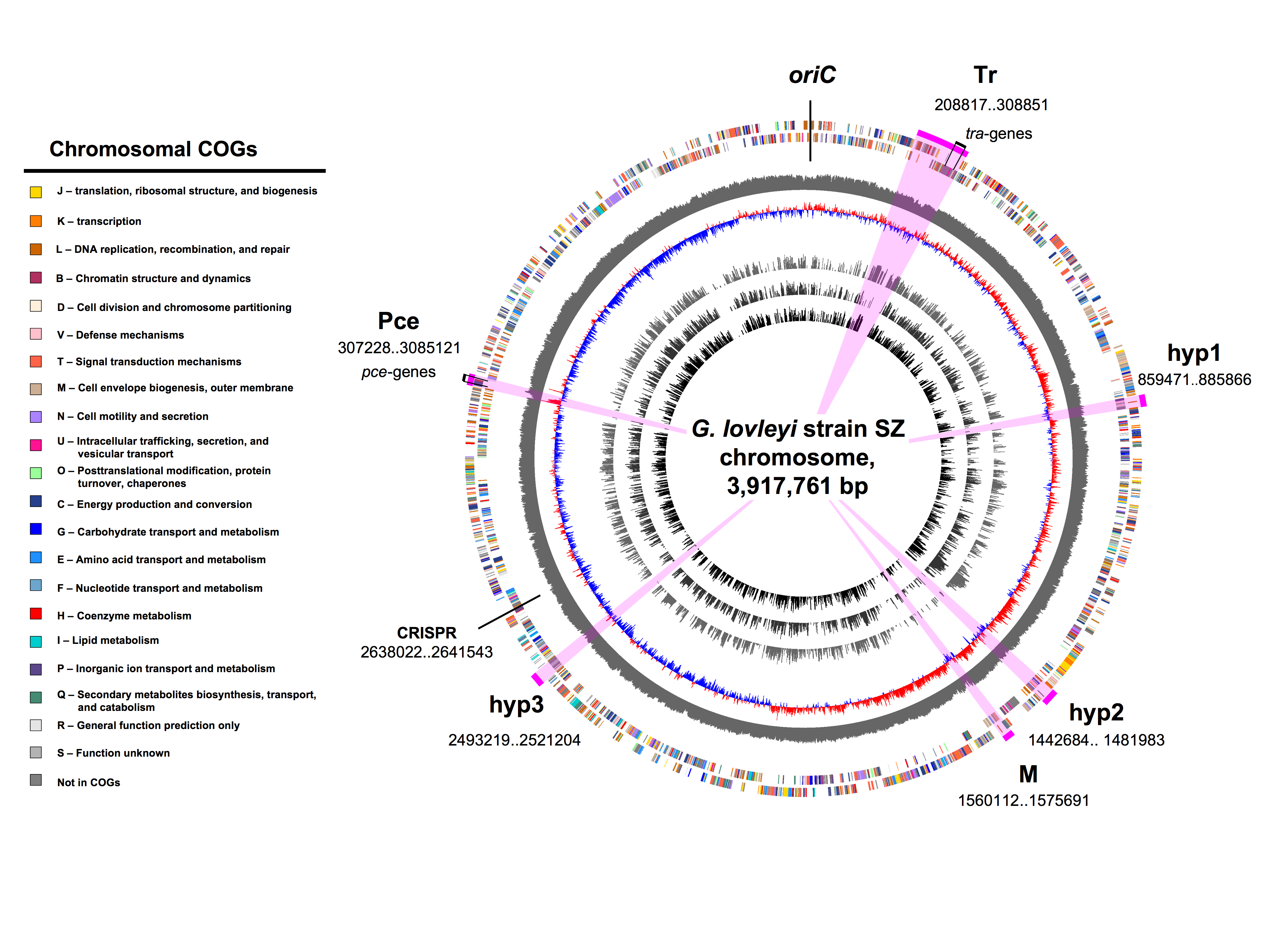


**Additional file 3:** Circular genome map of the *G. lovleyi* strain SZ chromosome (CP001089). From outside to center: COG categories of genes on forward strand, COG categories of genes on reverse strand, percent GC content, GC skew, and percent blastx identity of strain SZ ORFs to ORFs on the chromosomes of *Pelobacter propionicus* (CP000482), *G. uraniireducens*, and *G. sulfurreducens*. Genomic island regions, six in all, are indicated by pink arcs and are characterized by genes lacking blast matches in a majority of other *Geobacter*/*Pelobacter* spp. genomes (e.g. the three innermost circles), suggesting horizontal gene transfer. The genomic island Pce harbors the *pce*-gene cluster, encoding the PCE reductive dehalogenases, and exhibits nucleotide sequence deviations in GC% and GC-skew relative to the entire strain SZ chromosome. The shromosome map was generated using GenomeViz [81].
