# Supplementary material for: Genomic determinants of organohalide-respiration in Geobacter lovleyi, an unusual member of the Geobacteraceae
Source: BMC Genomics. 2012 May 22;13:200. doi: 10.1186/1471-2164-13-200 (PMC3403914; doi:10.1186/1471-2164-13-200)
Supplement: Additional file 4 — Codon usage of predicted F-factor conjugative pilustra-genes in comparison to all strain SZ chromosomal genes using the codon adaptation index (CAI). A majority of genes in the tra-pilus cluster have normalized CAI < 1.00 (red font), but are interspersed with genes having normalized CAI > 1.00, indicating sufficient residence time in strain SZ for the cluster to partially ameliorate to chromosomal codon usage. [file 1471-2164-13-200-S4.doc]

**Additional file 4:** Codon usage of predicted F-factor conjugative pilus *tra*-genes in comparison to all strain SZ chromosomal genes using the codon adaptation index (CAI). A majority of genes in the *tra*-pilus cluster have normalized CAI < 1.00 (red font), but are interspersed with genes having normalized CAI > 1.00, indicating sufficient residence time in strain SZ for the cluster to partially ameliorate to chromosomal codon usage.

| SZ chromosome locus | Function with predicted PFAM domains | Length (bp) | Normalized CAI |
| --- | --- | --- | --- |
| Glov_0304 | Conserved hypothetical protein | 324 | 0.85 |
| Glov_0305 | TraG – DNA transfer and F pilus assembly protein - pfam07916 | 3618 | 0.99 |
| Glov_0306 | Conserved hypothetical protein | 741 | 1.01 |
| Glov_0307 | Conserved hypothetical protein | 252 | 0.96 |
| Glov_0308 | TraF – type IV secretory protease – pfam10502 | 495 | 1.09 |
| Glov_0309 | TrbI – Conserved hypothetical protein – pfam09677 | 393 | 0.99 |
| Glov_0310 | TrbC – pilin assembly protein – pfam09673 | 963 | 1.02 |
| Glov_0311 | TraU – TraU family protein – pfam06834 | 1023 | 1.01 |
| Glov_0312 | Hypothetical protein, no NCBI database hits | 471 | 0.89 |
| Glov_0313 | TraW - Conserved hypothetical cytosolic protein (TraW – TIGR02743) | 657 | 0.98 |
| Glov_0314 | TraN - Hypothetical protein (TraN_Ftype – TIGR02750) | 3375 | 1.00 |
| Glov_0315 | Conserved hypothetical protein | 2004 | 1.07 |
| Glov_0316 | TraC – sex pilus assembly protein – pfam11130 | 2448 | 1.07 |
| Glov_0317 | TraV – conserved hypothetical protein – pfam09676 | 495 | 1.05 |
| Glov_0318 | TrbI – TraB pilus assembly family protein – (TrbI) pfam03743 | 1269 | 0.95 |
| Glov_0319 | TraK – conserved hypothetical protein – pfam06586 | 948 | 0.98 |
| Glov_0320 | TraE – conserved hypothetical sex pilus assembly and synthesis protein - pfam05309 | 579 | 1.08 |
| Glov_0321 | TraL – conserved hypothetical protein – pfam07178 | 267 | 0.97 |
| Glov_0322 | TrbC – conserved hypothetical protein – pfam04956 | 300 | 0.97 |
| * Normalized to codon usage over the entire strain SZ chromosome | | | |
