# Supplementary material for: Genomic determinants of organohalide-respiration in Geobacter lovleyi, an unusual member of the Geobacteraceae
Source: BMC Genomics. 2012 May 22;13:200. doi: 10.1186/1471-2164-13-200 (PMC3403914; doi:10.1186/1471-2164-13-200)
Supplement: Additional file 5 — Inferredc-type cytochrome genes on theG. lovleyistrain SZ chromosome. [file 1471-2164-13-200-S5.doc]

**Additional file 5:** Inferred *c*-type cytochrome genes on the *G. lovleyi* strain SZ chromosome.

| Locus | Gene symbol | CxxCH motifs | RefSeq ID of top BlastP match | Genome of top  BlastP match | % Ident. | Similarity* |
| --- | --- | --- | --- | --- | --- | --- |
| Glov_0202 |  | 2 | YP_383296 | *Geobacter metallireducens* GS-15 | 55 | 183/255 |
| Glov_0209 |  | 3 | YP_383303 | *Geobacter metallireducens* GS-15 | 74 | 72/93 |
| Glov_0211 | *nrfA* | 5 | ZP_05311341 | *Geobacter* sp. M18 | 72 | 375/445 |
| Glov_0642 |  | 2 | YP_001230785 | *Geobacter uraniireducens* Rf4 | 60 | 435/599 |
| Glov_0860 |  | 8 | YP_901261 | *Pelobacter propionicus* DSM 2379 | 71 | 400/470 |
| Glov_0887 |  | 4 | YP_001365373 | *Shewanella baltica* OS185 | 40 | 64/106 |
| Glov_0942 |  | 6 | YP_002135959 | *Anaeromyxobacter* sp. K | 58 | 58/84 |
| Glov_0946 |  | 4 | ZP_01313446 | *Desulfuromonas acetoxidans* DSM684 | 49 | 73/114 |
| Glov_1004 |  | 6 | YP_002135959 | *Anaeromyxobacter* sp. K | 56 | 61/85 |
| Glov_1042 | *nrfA* | 5 | YP_899549 | *Pelobacter propionicus* DSM 2379 | 70 | 384/462 |
| Glov_1043 | *nrfH* | 4 | YP_383265 | *Geobacter metallireducens* GS-15 | 60 | 115/147 |
| Glov_1044 |  | 8 | ZP_05311062 | *Geobacter* sp. M18 | 60 | 298/406 |
| Glov_1051 |  | 3 | YP_001232971 | *Geobacter uraniireducens* Rf4 | 71 | 52/63 |
| Glov_1059 |  | 2 | YP_004196830 | *Geobacter* sp. M18 | 54 | 64/92 |
| Glov_1061 |  | 7 | ZP_05311265 | *Geobacter* sp. M18 | 71 | 447/544 |
| Glov_1150 |  | 3 | YP_001229258 | *Geobacter uraniireducens* Rf4 | 58 | 70/101 |
| Glov_1172 |  | 5 | YP_383502 | *Geobacter metallireducens* GS-15 | 59 | 233/316 |
| Glov_1177 |  | 8 | YP_003020606 | *Geobacter* sp. M21 | 69 | 384/477 |
| Glov_1194 |  | 4 | YP_002536809 | *Geobacter* sp. FRC-32 | 46 | 148/251 |
| Glov_1198 | *mtrF* | 10 | YP_002140812 | *Geobacter bemidjiensis* Bem | 57 | 297/413 |
| Glov_1199 |  | 12 | ZP_05310895 | *Geobacter* sp. M18 | 60 | 456/593 |
| Glov_1201 |  | 5 | YP_001229236 | *Geobacter uraniireducens* Rf4 | 63 | 221/302 |
| Glov_1229 |  | 1 | NP_954374 | *Geobacter sulfurreducens* PCA | 51 | 55/81 |
| Glov_1291 |  | 6 | YP_003502960 | *Denitrovibrio acetiphilus* DSM 12809 | 63 | 108/139 |
| Glov_1315 |  | 12 | YP_003021711 | *Geobacter* sp. M21 | 57 | 237/333 |
| Glov_1467 |  | 9 | ZP_05310213 | *Geobacter* sp. M18 | 47 | 382/604 |
| Glov_1468 |  | 9 | ZP_05310213 | *Geobacter* sp. M18 | 47 | 404/642 |
| Glov_1483 |  | 1 | NP_952450 | *Geobacter sulfurreducens* PCA | 70 | 473/561 |
| Glov_1703 |  | 12 | NP_951650 | *Geobacter sulfurreducens* PCA | 50 | 200/324 |
| Glov_1706 |  | 5 | YP_001232378 | *Geobacter uraniireducens* Rf4 | 52 | 119/175 |
| Glov_1710 |  | 5 | YP_900554 | *Pelobacter propionicus* DSM 2379 | 61 | 379/490 |
| Glov_1719 |  | 8 | YP_901261 | *Pelobacter propionicus* DSM 2379 | 72 | 390/471 |

**Additional file 5:**  (Continued)

| Locus | Gene symbol | CxxCH motifs | RefSeq ID of top BlastP match | Genome of top  BlastP match | % Ident. | Similarity* |
| --- | --- | --- | --- | --- | --- | --- |
| Glov_1762 |  | 3 | ZP_01312357 | *Desulfuromonas acetoxidans* DSM684 | 49 | 372/571 |
| Glov_1764 |  | 8 | YP_003020606 | *Geobacter* sp. M21 | 66 | 376/480 |
| Glov_1803 |  | 6 | ZP_05313793 | *Geobacter* sp. M18 | 70 | 318/393 |
| Glov_1903 |  | 1 | YP_001230676 | *Geobacter uraniireducens* Rf4 | 53 | 46/69 |
| Glov_2063 | *mtrA* | 9 | YP_001228914 | *Geobacter uraniireducens* Rf4 | 66 | 465/604 |
| Glov_2292 | *mtrF* | 10 | ZP_05313572 | *Geobacter* sp. M18 | 43 | 473/835 |
| Glov_2294 |  | 8 | YP_002140171 | *Geobacter bemidjiensis* Bem | 57 | 178/245 |
| Glov_2295 |  | 9 | YP_002537141 | *Geobacter* sp. FRC-32 | 38 | 283/575 |
| Glov_2299 | *mtrF* | 10 | YP_525308 | *Rhodoferax ferrireducens* T118 | 52 | 267/396 |
| Glov_2651 |  | 1 | YP_383296 | *Geobacter metallireducens* GS-15 | 33 | 69/143 |
| Glov_2758 |  | 3 | YP_383303 | *Geobacter metallireducens* GS-15 | 52 | 57/94 |
| Glov_2825 |  | 9 | YP_002138815 | *Geobacter bemidjiensis* Bem | 71 | 503/607 |
| Glov_2826 |  | 2 | YP_901075 | *Pelobacter propionicus* DSM 2379 | 57 | 75/101 |
| Glov_3048 | *mtrF* | 10 | ZP_05310497 | *Geobacter* sp. M18 | 43 | 406/716 |
| Glov_3459 |  | 2 | YP_903254 | *Pelobacter propionicus* DSM 2379 | 61 | 86/111 |
| Glov_3541 |  | 4 | ZP_05311743 | *Geobacter* sp. M18 | 72 | 481/577 |
| Glov_3625 |  | 4 | YP_002140822 | *Geobacter bemidjiensis* Bem | 80 | 509/576 |
| *Similarity shown as a fraction of positives to aligned length of query to BlastP match | | | | | | |
