# Supplementary material for: Genomic determinants of organohalide-respiration in Geobacter lovleyi, an unusual member of the Geobacteraceae
Source: BMC Genomics. 2012 May 22;13:200. doi: 10.1186/1471-2164-13-200 (PMC3403914; doi:10.1186/1471-2164-13-200)
Supplement: Additional file 6 — Inferred c-type cytochrome genes inPelobacter propionicusDSM 2379. [file 1471-2164-13-200-S6.doc]

**Additional file 6:** Inferred *c*-type cytochrome genes in *Pelobacter propionicus* DSM 2379.

| Locus | Gene symbol | CxxCH motifs | RefSeq ID of top BlastP match | Genome of top  BlastP match | % Ident. | Similarity |
| --- | --- | --- | --- | --- | --- | --- |
| **Chromosome** | | | | | | |
| Ppro_0721 |  | 1 | YP_385520 | *Geobacter metallireducens* GS-15 | 75 | 144/163 |
| Ppro_0828 |  | 1 | NP_954314 | *Geobacter sulfurreducens* PCA | 67 | 112/137 |
| Ppro_0867 |  | 5 | YP_383646 | *Geobacter metallireducens* GS-15 | 62 | 365/470 |
| Ppro_1199 | *nrfH* | 4 | YP_001951286 | *Geobacter lovleyi* SZ | 66 | 122/153 |
| Ppro_1200 | *nrfA* | 5 | YP_383264 | *Geobacter metallireducens* GS-15 | 70 | 387/468 |
| Ppro_1401 |  | 2 | YP_001953059 | *Geobacter lovleyi* SZ | 57 | 75/101 |
| Ppro_1575 |  | 18 | YP_001231869 | *Geobacter uraniireducens* Rf4 | 44 | 269/465 |
| Ppro_1577 |  | 7 | YP_385871 | *Geobacter metallireducens* GS-15 | 54 | 252/366 |
| Ppro_1588 |  | 8 | YP_001951104 | *Geobacter lovleyi* SZ | 71 | 400/470 |
| Ppro_1603 |  | 1 | YP_001951979 | *Geobacter lovleyi* SZ | 60 | 117/155 |
| Ppro_1916 |  | 1 | YP_958531 | *Marinobacter aquaeolei* VT8 | 99 | 157/157 |
| Ppro_2999 |  | 7 | YP_004199549 | *Geobacter* sp. M18 | 77 | 477/537 |
| Ppro_3498 |  | 6 | YP_001952039 | *Geobacter lovleyi* SZ | 67 | 310/389 |
| Ppro_3509 |  | 3 | YP_383303 | *Geobacter metallireducens* GS-15 | 67 | 67/86 |
| Ppro_3605 |  | 2 | YP_001953682 | *Geobacter lovleyi* SZ | 61 | 86/111 |
| **Plasmid pPRO1** | | | | | | |
| Ppro_3655 |  | 1 | YP_002538020 | *Geobacter* sp. FRC-32 | 56 | 106/139 |
| Ppro_3705 | *nrfH* | 5 | YP_001229450 | *Geobacter uraniireducens* Rf4 | 80 | 417/463 |
| Ppro_3707 | *nrfA* | 4 | YP_001229449 | *Geobacter uraniireducens* Rf4 | 70 | 127/152 |
| **Plasmid pPRO2** | | | | | | |
| Ppro_3831 |  | 1 | YP_958531 | *Marinobacter aquaeolei* VT8 | 99 | 157/157 |
| Ppro_3848 |  | 1 | YP_958531 | *Marinobacter aquaeolei* VT8 | 99 | 157/157 |
