# Supplementary material for: Genomic determinants of organohalide-respiration in Geobacter lovleyi, an unusual member of the Geobacteraceae
Source: BMC Genomics. 2012 May 22;13:200. doi: 10.1186/1471-2164-13-200 (PMC3403914; doi:10.1186/1471-2164-13-200)
Supplement: Additional file 7 — Electron-transfer proteins encoded on theG. lovleyistrain SZ chromosome. [file 1471-2164-13-200-S7.doc]

**Additional file 7:**  Electron-transfer proteins encoded on the *G. lovleyi* strain SZ chromosome.

| Locus | Function | RefSeq ID of top BlastP match | Genome of top  BlastP match | % Ident. | Similarity |
| --- | --- | --- | --- | --- | --- |
| **Proteins containing molybdopterin oxidoreductase-domain (pfam00384 - underlined) and associated respiratory-type proteins** | | | | | |
| Glov_0203 | Nitrate reductase, alpha subunit | YP_383297 | *Geobacter metallireducens* GS-15 | 83 | 1082/1191 |
| Glov_0204 | Nitrate reductase, beta subunit | YP_383298 | *Geobacter metallireducens* GS-15 | 78 | 417/483 |
| Glov_0205 | Nitrate reductase molybdenum cofactor assembly | YP_383299 | *Geobacter metallireducens* GS-15 | 58 | 121/172 |
| Glov_0206 | Respiratory nitrate reductase, gamma subunit | YP_383300 | *Geobacter metallireducens* GS-15 | 71 | 186/221 |
| Glov_0661 | Molybdopterin oxidoreductase | YP_002137483 | *Geobacter bemidjiensis* Bem | 51 | 545/823 |
| Glov_0899 | Formate dehydrogenase | ZP_01311645 | *Desulfuromonas acetoxidans* DSM 684 | 79 | 669/753 |
| Glov_0900 | Iron-sulfur oxidoreductase | YP_519758 | *Desulfitobacterium hafniense* Y51 | 72 | 97/111 |
| Glov_0930 | Molybdopterin oxidoreductase | YP_519751 | *Desulfitobacterium hafniense* Y51 | 63 | 559/722 |
| Glov_0933 | NrfD polysulfide reductase | ZP_01311643 | *Desulfuromonas acetoxidans* DSM 684 | 58 | 237/314 |
| Glov_0934 | 4Fe-4S ferredoxin | ZP_01311642 | *Desulfuromonas acetoxidans* DSM 684 | 72 | 148/181 |
| Glov_0935 | Molybdopterin oxidoreductase | ZP_01311641 | *Desulfuromonas acetoxidans* DSM 684 | 78 | 671/747 |
| Glov_1055 | NapD family | YP_001767985 | *Methylobacterium* sp. 4-46 | 32 | 42/73 |
| Glov_1056 | Periplasmic nitrate reductase | ZP_01105283 | *Flavobacteriales bacterium* HTCC2170 | 53 | 535/773 |
| Glov_1057 | NapG ferredoxin | YP_001500485 | *Shewanella pealeana* ATCC 700345 | 48 | 136/199 |
| Glov_1058 | NapH ferredoxin | YP_732839 | *Shewanella* sp. MR-4 | 43 | 156/249 |
| Glov_1148 | 4Fe-4S ferredoxin | ZP_03904963 | *Denitrovibrio acetiphilus* DSM 12809 | 62 | 182/250 |
| Glov_1149 | Molybdopterin oxidoreductase | YP_001229259 | *Geobacter uraniireducens* Rf4 | 80 | 761/867 |
| Glov_1162 | NrfD polysulfide reductase | YP_002248979 | *Thermodesulfovibrio yellowstonii* DSM 11347 | 46 | 221/327 |
| Glov_1163 | 4Fe-4S ferredoxin | YP_002248980 | *Thermodesulfovibrio yellowstonii* DSM 11347 | 61 | 146/181 |
| Glov_1164 | Formate dehydrogenase | YP_002248981 | *Thermodesulfovibrio yellowstonii* DSM 11347 | 58 | 546/727 |
| Glov_1443 | Molybdopterin oxidoreductase | ZP_03735695 | *Desulfonatronospira thiodismutans* ASO3-1 | 67 | 330/410 |

**Additional file 7:**  (continued)

| Locus | Function | RefSeq ID of top BlastP match | Genome of top  BlastP match | % Ident. | Similarity |
| --- | --- | --- | --- | --- | --- |
| **Proteins containing molybdopterin oxidoreductase-domain (pfam00384 - underlined) and associated respiratory-type proteins (continued)** | | | | | |
| Glov_2652 | Nitrate reductase, alpha subunit | YP_383297 | *Geobacter metallireducens* GS-15 | 76 | 1044/1193 |
| Glov_2653 | Nitrate reductase, beta subunit | YP_001950455 | *Geobacter lovleyi* SZ | 71 | 404/481 |
| Glov_2654 | Nitrate reductase molybdenum cofactor assembly | YP_383299 | *Geobacter metallireducens* GS-15 | 52 | 114/173 |
| Glov_2655 | Respiratory nitrate reductase, gamma subunit | YP_383300 | *Geobacter metallireducens* GS-15 | 59 | 169/227 |
| Glov_3550 | Molybdopterin oxidoreductase | YP_001230059 | *Geobacter uraniireducens* Rf4 | 60 | 491/661 |
| **Proteins containing flavoprotein domain (pfam00890 - underlined) and associated respiratory-type proteins** | | | | | |
| Glov_0943 | Flavocytochrome c | YP_002460220 | *Desulfitobacterium hafniense* DCB-2 | 53 | 358/517 |
| Glov_0945 | Succinate dehydrogenase | ZP_01313445 | *Desulfuromonas acetoxidans* DSM 684 | 68 | 413/509 |
| Glov_0946 | Fumarate reductase flavoprotein subunit | ZP_01313446 | *Desulfuromonas acetoxidans* DSM 684 | 50 | 73/111 |
| Glov_1003 | Fumarate reductase/succinate dehydrogenase | YP_002554243 | *Diaphorobacter* sp. TPSY | 31 | 238/531 |
| Glov_1298 | Fumarate reductase/succinate dehydrogenase | YP_002457273 | *Desulfitobacterium hafniense* DCB-2 | 31 | 274/583 |
| Glov_2212 | 4Fe-4S ferredoxin | YP_902144 | *Pelobacter propionicus* DSM 2379 | 84 | 228/251 |
| Glov_2213 | Succinate dehydrogenase flavoprotein | YP_001231990 | *Geobacter uraniireducens* Rf4 | 90 | 608/638 |
| Glov_2214 | Succinate dehydrogenase, cytochrome b | YP_902146 | *Pelobacter propionicus* DSM 2379 | 60 | 171/222 |
| Glov_3540 | C4-dicarboxylate antiporter (Dcu) | ZP_03025442 | *Geobacter* sp. M21 | 88 | 414/439 |
| Glov_3541 | Flavocytochrome c | YP_002140385 | *Geobacter bemidjiensis* Bem | 72 | 481/583 |
| Glov_3625 | Flavocytochrome c | YP_002140822 | *Geobacter bemidjiensis* Bem | 79 | 518/596 |

**Additional file 7:**  (continued)

| Locus | Function | RefSeq ID of top BlastP match | Genome of top  BlastP match | % Ident. | Similarity |
| --- | --- | --- | --- | --- | --- |
| **Components to pyruvate flavodoxin/ferredoxin oxidoreductase (PFOR) complexes** | | | | | |
| Glov_0368 | Pyruvate flavodoxin/ferredoxin oxidoreductase domain protein | NP_951159 | *Geobacter sulfurreducens* PCA | 80 | 1067/1195 |
| Glov_1075 | Thiamine pyrophosphate protein domain protein | ZP_03856815 | *Thermobaculum terrenum* ATCC BAA-798 | 63 | 269/332 |
| Glov_1076 | Pyruvate flavodoxin/ferredoxin oxidoreductase domain protein | YP_001432409 | *Roseiflexus castenholzii* DSM 13941 | 63 | 460/592 |
| Glov_1627 | Pyruvate flavodoxin/ferredoxin oxidoreductase domain protein | YP_001230951 | *Geobacter uraniireducens* Rf4 | 80 | 341/377 |
| Glov_1628 | Thiamine pyrophosphate protein domain protein | YP_384322 | *Geobacter metallireducens* GS-15 | 91 | 264/272 |
| Glov_1629 | Pyruvate ferredoxin/flavodoxin oxidoreductase | NP_952521 | *Geobacter sulfurreducens* PCA | 80 | 159/177 |
| Glov_2307 | Pyruvate ferredoxin/flavodoxin oxidoreductase | YP_384270 | *Geobacter metallireducens* GS-15 | 72 | 150/175 |
| Glov_2308 | Thiamine pyrophosphate protein domain protein TPP-binding | YP_001230994 | *Geobacter uraniireducens* Rf4 | 80 | 222/247 |
| Glov_2309 | Pyruvate flavodoxin/ferredoxin oxidoreductase domain protein | YP_002538449 | *Geobacter* sp. FRC-32 | 76 | 302/347 |
| Glov_3719 on pSZ77 | Thiamine pyrophosphate protein domain protein | YP_158793 | *Aromatoleum aromaticum* EbN1 | 51 | 232/330 |
| Glov_3720 on pSZ77 | Pyruvate flavodoxin/ferredoxin oxidoreductase domain protein | YP_290730 | *Thermobifida fusca* YX | 54 | 432/608 |

**Additional file 7:**  (continued)

| Locus | Function | RefSeq ID of top BlastP match | Genome of top  BlastP match | % Ident. | Similarity |
| --- | --- | --- | --- | --- | --- |
| **Proteins containing nickel-dependent hydrogenases (pfam00374 - underlined) and associated respiratory-type proteins** | | | | | |
| Glov_0144 | Hydrogenase small subunit HydA | YP_001230706 | *Geobacter uraniireducens* Rf4 | 79 | 328/370 |
| Glov_0145 | 4Fe-4S ferredoxin | YP_001230707 | *Geobacter uraniireducens* Rf4 | 76 | 267/308 |
| Glov_0146 | Polysulphide reductase NrfD | YP_002139935 | *Geobacter bemidjiensis* Bem | 73 | 356/412 |
| Glov_0147 | Nickel-dependent hydrogenase | NP_951842 | *Geobacter sulfurreducens* PCA | 78 | 493/557 |
| Glov_1552 | NADH ubiquinone oxidoreductase 20 kDa sub | ZP_03025588 | *Geobacter* sp. M21 | 73 | 272/308 |
| Glov_1553 | Nickel-dependent hydrogenase | YP_002140672 | *Geobacter bemidjiensis* Bem | 69 | 398/472 |
| Glov_1554 | 4Fe-4S ferredoxin | YP_002140671 | *Geobacter bemidjiensis* Bem | 79 | 74/82 |
| Glov_2054 | Hydrogenase cytochrome b subunit | YP_001229653 | *Geobacter uraniireducens* Rf4 | 57 | 153/210 |
| Glov_2055 | Nickel-dependent hydrogenase | YP_001229331 | *Geobacter uraniireducens* Rf4 | 82 | 514/565 |
| Glov_2791 | Nickel-dependent hydrogenase | YP_846829 | *Syntrophobacter fumaroxidans* MPOB | 61 | 370/478 |
| Glov_2792 | NADH ubiquinone oxidoreductase, 20 kDa | YP_846828 | *Syntrophobacter fumaroxidans* MPOB | 57 | 132/178 |
| Glov_2793 | Ferredoxin | YP_384076 | *Geobacter metallireducens* GS-15 | 62 | 182/231 |
| Glov_2794 | NADH dehydrogenase | YP_846826 | *Syntrophobacter fumaroxidans* MPOB | 65 | 440/545 |
| Glov_2795 | NADH dehydrogenase, 24 kDa | YP_384074 | *Geobacter metallireducens* GS-15 | 61 | 117/155 |
