# Supplementary material for: Genomic determinants of organohalide-respiration in Geobacter lovleyi, an unusual member of the Geobacteraceae
Source: BMC Genomics. 2012 May 22;13:200. doi: 10.1186/1471-2164-13-200 (PMC3403914; doi:10.1186/1471-2164-13-200)
Supplement: Additional file 8 — Inferred reactive-oxygen-species-responsive or oxygen-reducing genes inG. lovleyistrain SZ. [file 1471-2164-13-200-S8.doc]

**Additional file 8:**  Inferred reactive-oxygen-species-responsive or oxygen-reducing genes in *G. lovleyi* strain SZ.

| Locus | Function | RefSeq ID of top BlastP match | Genome of top  BlastP match | % Ident. | Similarity |
| --- | --- | --- | --- | --- | --- |
| Glov_0804 | Rubrerythrin | NP_951443 | *Geobacter sulfurreducens* PCA | 68 | 128/154 |
| Glov_0828 | Rubrerythrin | NP_954329 | *Geobacter sulfurreducens* PCA | 68 | 135/165 |
| Glov_1052 | Rubrerythrin | YP_386180 | *Geobacter metallireducens* GS-15 | 52 | 110/149 |
| Glov_1208 | Cytochrome bd ubiquinol oxidase subunit I | YP_384884 | *Geobacter metallireducens* GS-15 | 70 | 371/447 |
| Glov_1209 | Cytochrome d ubiquinol oxidase subunit II | NP_952692 | *Geobacter sulfurreducens* PCA | 74 | 284/340 |
| Glov_1794 | Rubrerythrin | YP_001232869 | *Geobacter uraniireducens* Rf4 | 77 | 149/168 |
| Glov_1795 | Catalase/peroxidase HPI | YP_356594 | *Pelobacter carbinolicus* DSM 2380 | 82 | 656/735 |
| Glov_1797 | Rubrerythrin | YP_901632 | *Pelobacter propionicus* DSM 2379 | 83 | 168/189 |
| Glov_3343 | Ferredoxin | YP_386108 | *Geobacter metallireducens* GS-15 | 67 | 52/62 |
| Glov_3344 | Rubredoxin | ZP_03014104 | *Bacteroides intestinalis* DSM 17393 | 68 | 41/50 |
| Glov_0804 | Rubrerythrin | NP_951443 | *Geobacter sulfurreducens* PCA | 68 | 128/154 |
