# Supplementary material for: Genomic determinants of organohalide-respiration in Geobacter lovleyi, an unusual member of the Geobacteraceae
Source: BMC Genomics. 2012 May 22;13:200. doi: 10.1186/1471-2164-13-200 (PMC3403914; doi:10.1186/1471-2164-13-200)
Supplement: Additional file 9 — Circular genome map of theG. lovleyistrain SZ plasmid pSZ77 (CP001090). From outside to center: COG categories of genes on forward strand, COG categories of genes on reverse strand, percent GC content, GC skew, percent blastx identity of SZ ORFs to plasmids of Pelobacter propionicus (CP000483 and CP000484), and percent blastx identity to the chromosomes of Pelobacter propionicus (CP000482), Geobacter uraniireducens, and G. sulfurreducens. The plasmid map was generated using Genome Viz [81]. [file 1471-2164-13-200-S9.doc]

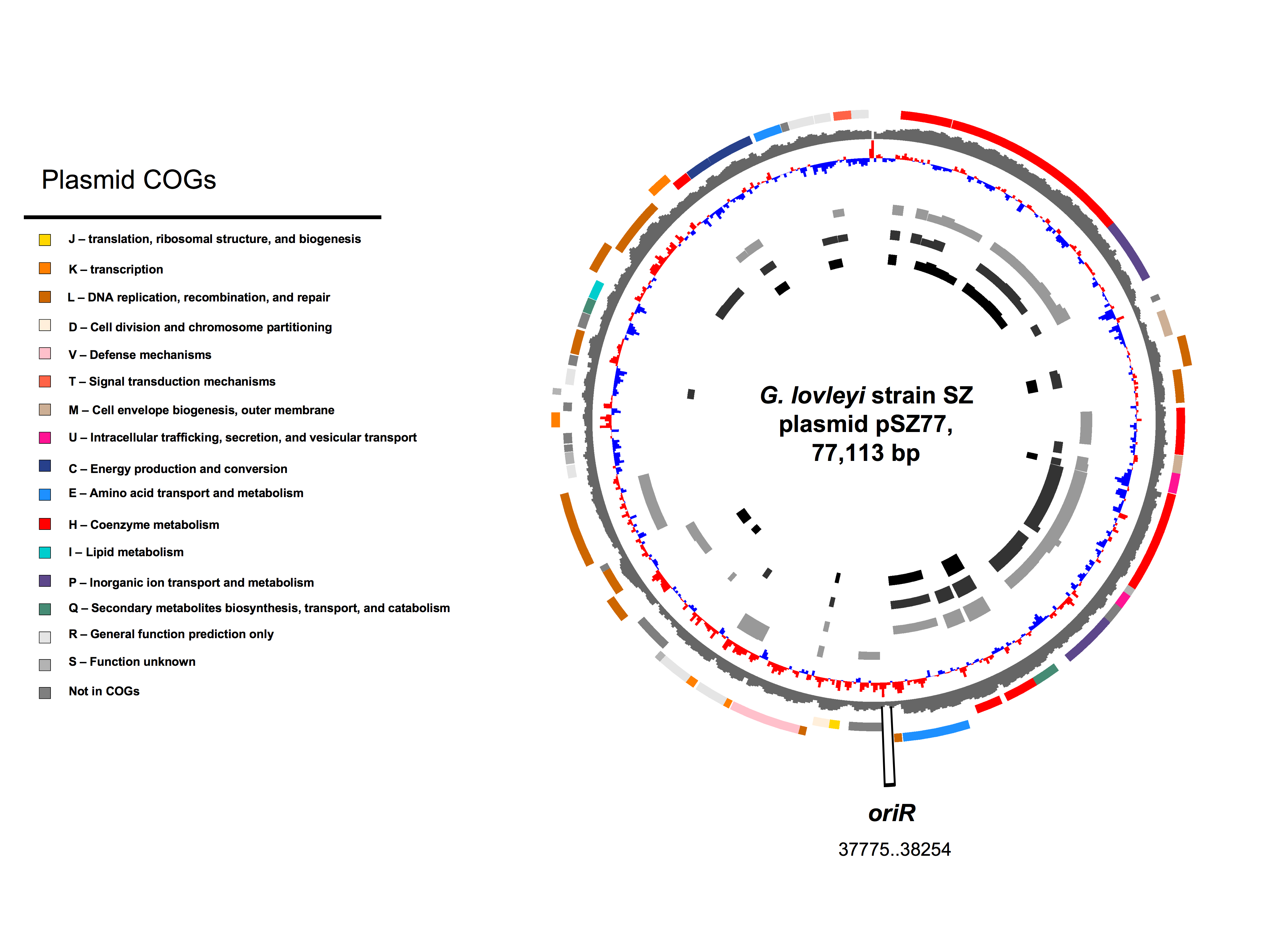


**Additional file 9:** Circular genome map of the *G. lovleyi* strain SZ plasmid pSZ77 (CP001090). From outside to center: COG categories of genes on forward strand, COG categories of genes on reverse strand, percent GC content, GC skew, percent blastx identity of SZ ORFs to plasmids of *Pelobacter propionicus* (CP000483 and CP000484), and percent blastx identity to the chromosomes of *Pelobacter propionicus* (CP000482), *Geobacter uraniireducens*, and *G. sulfurreducens*. The plasmid map was generated using GenomeViz [81].
