# Supplementary material for: Genomic determinants of organohalide-respiration in Geobacter lovleyi, an unusual member of the Geobacteraceae
Source: BMC Genomics. 2012 May 22;13:200. doi: 10.1186/1471-2164-13-200 (PMC3403914; doi:10.1186/1471-2164-13-200)
Supplement: Additional file 10 — pSZ77 genes related to plasmid replication, maintenance, and recombination. [file 1471-2164-13-200-S10.doc]

**Additional file 10:** pSZ77 genes related to plasmid replication, maintenance, and recombination.

| pSZ77 locus | Gene symbol | Annotation | Best match to, % identity (similarity) | | |
| --- | --- | --- | --- | --- | --- |
|  |  |  | SZ chromosome | *Geobacter* genomes | *Pelobacter*  genomes |
| Glov_3681 | *repA* | Plasmid replication protein | None | *G. metallireducens*  14 kbp plasmid  33% (153/303) | *P. propionicus*  31 kbp plasmid 35% (166/293) |
| Glov_3684* | *parA* | Partitioning ATPase | None | None | None |
| Glov_3687 | *hipB* | Transcriptional regulator, XRE family | None | *Geobacter* sp. M21 chromosome  64% (77/78) | *P. propionicus* 202 kbp plasmid  73% (66/78) |
| Glov_3688 | *hipA* | HipA domain protein | None | *Geobacter* sp.  FRC-32 chromosome 28% (159/346) | *P. propionicus* 202 kbp plasmid  75% (344/399) |
| Glov_3689 | *hipB?* | Transcriptional regulator, XRE family | Glov_0180  31% (42/83) | *G. uraniireducens* chromosome 53% (77/112) | *P. propionicus* chromosome 28% (52/100) |
| Glov_3690 | *hipA* | HipA domain protein | Glov_0325 30% (173/406) | *Geobacter* sp.  M18 chromosome  65% (331/412) | *P. propionicus* chromosome  29% (168/383) |
| Glov_3698 | *Tn3* | Transposase Tn3 | None | None | *P. propionicus* 202 kbp plasmid  46% (640/977) |
| Glov_3709 | *-* | Integrase | Glov_0344 100% | None | *P. carbinolicus* chromosome  82% (292/324) |
| Glov_3713 | *-* | Transposase | Glov_2893 100% | None | *P. carbinolicus* chromosome  46% (53/92) |
| Glov_3714 | *-* | Integrase | Glov_2892 100% | None | None |
| * The putative pSZ77 ParA protein shares 42% aa identity (61% similarity) with its most similar homolog, a putative ParA on plasmid pMRAD08 from *Methylobacterium radiotolerans* JCM 2831 | | | | | |
