# Supplementary material for: Genomic determinants of organohalide-respiration in Geobacter lovleyi, an unusual member of the Geobacteraceae
Source: BMC Genomics. 2012 May 22;13:200. doi: 10.1186/1471-2164-13-200 (PMC3403914; doi:10.1186/1471-2164-13-200)
Supplement: Additional file 11 — Inferred cobalamin-dependent genes on theG. lovleyistrain SZ chromosome. [file 1471-2164-13-200-S11.doc]

**Additional file 11:** Inferred cobalamin-dependent genes on the *G. lovleyi* strain SZ chromosome.

| Locus | Function | RefSeq ID of top BlastP match | Genome of top  BlastP match | % Ident. | Similarity |
| --- | --- | --- | --- | --- | --- |
| Glov_2133 | Methionine synthase | ZP_08643025 | *Brevibacillus laterosporus* | 60% | 872/1161 |
| Glov_2318 | Ribonucleotide-diphosphate reductase | YP_902161 | *Pelobacter propionicus* DSM 2379 | 80% | 660/735 |
| Glov_2780 | PCE reductive dehalogenase | YP_519072 | *Desulfitobacterium hafniense* strain Y51 (strain TCE1) | 36%  (34%) | 313/552  (308/550) |
| Glov_2782 | PCE reductive dehalogenase | YP_519072 | *Desulfitobacterium hafniense* Y51  (strain TCE1) | 35%  (34%) | 311/552  (308/550) |
| Glov_3260 | Methylmalonyl-CoA mutase | YP_003504649 | *Denitrovibrio acetiphilus* DSM 12809 | 79% | 642/712 |
| Glov_0797 | Radical SAM domain protein | YP_003657093 | *Arcobacter nitrofigilis* DSM 7299 | 58% | 364/484 |
| Glov_1384 | Transcriptional regulator, MerR family | YP_003169177 | *Candidatus Accumulibacter phosphatis* | 41% | 165/275 |
| Glov_2185 | Radical SAM domain protein | YP_384661 | *Geobacter metallireducens* GS-15 | 74% | 397/473 |
| Glov_2195 | Radical SAM domain protein | NP_951299 | *Geobacter sulfurreducens* PCA | 64% | 361/459 |
| Glov_2203 | Cobalamin B12-binding domain protein | NP_951308 | *Geobacter sulfurreducens* PCA | 76% | 411/471 |
| Glov_2204 | Radical SAM domain protein | YP_384656 | *Geobacter metallireducens* GS-15 | 74% | 360/419 |
| Glov_2205 | Radical SAM domain protein | NP_951302 | *Geobacter sulfurreducens* PCA | 74% | 373/423 |
| Glov_2992 | Radical SAM domain protein | YP_383075 | *Geobacter metallireducens* GS-15 | 50% | 402/620 |
| Glov_3057 | Radical SAM domain protein | YP_902300 | *Pelobacter propionicus* DSM 2379 | 48% | 335/516 |
| Glov_3090 | Hopanoid biosynthesis associated radical SAM protein | YP_902481 | *Pelobacter propionicus* DSM 2379 | 87% | 445/472 |
| Glov_3359 | Radical SAM domain protein | YP_356553 | *Pelobacter carbinolicus* DSM 2380 | 32% | 228/450 |
| Glov_3363 | Radical SAM domain protein | YP_004200859 | *Geobacter* sp. M18 | 51% | 538/824 |
| Glov_3401 | Radical SAM domain protein | YP_004012404 | *Rhodomicrobium vannielii* ATCC 17100 | 28% | 179/387 |
