# Supplementary material for: Genomic determinants of organohalide-respiration in Geobacter lovleyi, an unusual member of the Geobacteraceae
Source: BMC Genomics. 2012 May 22;13:200. doi: 10.1186/1471-2164-13-200 (PMC3403914; doi:10.1186/1471-2164-13-200)
Supplement: Additional file 12 — Codon usage of pSZ77 genes in comparison to strain SZ chromosomal genes using the codon adaptation index (CAI). Out of 79 total predicted plasmid genes, 42 have normalized CAI > 1.00, indicating that over half of pSZ77 genes have resided in the strain SZ genome for sufficient time to ameliorate their codon usage to the chromosome. The predicted replication/maintenance genes repA and parA have normalized CAI < 1.00 (red font) [82]. [file 1471-2164-13-200-S12.doc]

**Additional file 12:.** Codon usage of pSZ77 genes in comparison to strain SZ chromosomal genes using the codon adaptation index (CAI). Out of 79 total predicted plasmid genes, 42 have normalized CAI > 1.00, indicating that over half of pSZ77 genes have resided in the strain SZ genome for sufficient time to ameliorate their codon usage to the chromosome. The predicted replication/maintenance genes *repA* and *parA* have normalized CAI < 1.00 (red font) [82].

| pSZ77 locus | Inferred/Annotated Function | Length (bp) | Normalized CAI |
| --- | --- | --- | --- |
| Glov_3646 | Precorrin-6y C5,15-methyl-transferase, CbiE | 618 | 1.05 |
| Glov_3647 | Precorrin-6x reductase, CbiJ/CobK | 786 | 1.07 |
| Glov_3648 | Precorrin-3B C17-methyltransferase, CbiH | 669 | 1.15 |
| Glov_3649 | Cobalamin biosynthesis protein, CbiG | 780 | 1.02 |
| Glov_3650 | Precorrin-4 C11-methyltransferase, CbiF | 759 | 1.05 |
| Glov_3651 | Cobalamin biosynthesis protein, CbiD | 1077 | 1.09 |
| Glov_3652 | Precorrin-2 C20-methyltransferase, CbiL/CobI | 738 | 1.14 |
| Glov_3653 | Anaerobic cobalt chelatase, CbiK | 786 | 1.07 |
| Glov_3654 | Precorrin-8X methylmutase, CbiC/CobH | 687 | 1.08 |
| Glov_3655 | Cobyrinic acid a,c-diamide synthase, CbiA/CobB | 1443 | 1.11 |
| Glov_3656 | Fused siroheme synthase (SirA);  Uroporphyrin-III C-methyltransferase (CysG) | 1413 | 1.06 |
| Glov_3657 | Cobalt ABC transporter, ATPase subunit | 861 | 1.04 |
| Glov_3658 | Cobalt ABC transporter, inner membrane subunit | 729 | 0.94 |
| Glov_3659 | Cobalt transport protein, CbiN | 333 | 0.93 |
| Glov_3660 | Cobalt transport protein, CbiM | 678 | 1.00 |
| Glov_3661 | Hypothetical protein | 228 | 0.87 |
| Glov_3663 | RND family efflux transporter | 1032 | 0.85 |
| Glov_3664 | S3/IS911 family transposase | 288 | 0.96 |
| Glov_3665 | Integrase/Transposase | 918 | 1.04 |
| Glov_3666 | Transposase IS4 | 1329 | 1.09 |
| Glov_3667 | TonB-dependent receptor | 1875 | 1.04 |
| Glov_3668 | TonB family protein | 735 | 0.95 |
| Glov_3669 | Biopolymer transport protein ExbD/TolR | 384 | 0.84 |
| Glov_3670 | TonB system energizer ExbB | 435 | 0.88 |
| Glov_3671 | Aerobic cobaltochelatase | 4101 | 1.15 |
| Glov_3672 | Uncharacterized domain protein, DUF2149 | 339 | 1.15 |
| Glov_3673 | MotA/TolQ/ExbB proton channel | 597 | 1.13 |
| Glov_3674 | Uncharacterized domain protein, DUF2162 | 702 | 1.15 |
| Glov_3675 | TonB-dependent receptor | 2214 | 1.10 |
| Glov_3676 | O-methyltransferase family protein | 1035 | 1.11 |
| Glov_3677 | Hydroxymethyl-pyrimidine synthase, thiC | 1311 | 1.26 |

**Table S9.** (Continued)

| pSZ77 locus | Inferred/Annotated Function | Length (bp) | Normalized CAI |
| --- | --- | --- | --- |
| Glov_3678 | Nicotinate-nucleotide—DMBA phosphoribosyltransferase | 1053 | 1.22 |
| Glov_3679 | Fused L-threonine decarboxylase; cobyric acid synthase | 2592 | 1.17 |
| Glov_3680 | Histone-like protein | 294 | 0.78 |
| Glov_3681 | Replication initiation protein, RepA | 1113 | 0.89 |
| Glov_3682 | Hypothetical, no NCBI database hits | 195 | 0.88 |
| Glov_3683 | Hypothetical, no NCBI database hits | 423 | 0.91 |
| Glov_3684 | Plasmid partitioning ATPase, ParA | 651 | 0.98 |
| Glov_3685 | Histone family protein | 282 | 0.79 |
| Glov_3686 | Conserved hypothetical protein | 2757 | 1.04 |
| Glov_3687 | XRE family transcriptional regulator | 240 | 0.99 |
| Glov_3688 | HipA N-terminal domain protein | 1212 | 0.98 |
| Glov_3689 | XRE transcriptional regulator | 354 | 1.01 |
| Glov_3690 | HipA domain protein | 1245 | 0.98 |
| Glov_3691 | WGR domain protein | 255 | 1.08 |
| Glov_3692 | Hypothetical, no NCBI database hits | 705 | 0.95 |
| Glov_3693 | Hypothetical | 510 | 0.90 |
| Glov_3694 | Hypothetical, no NCBI database hits | 225 | 0.87 |
| Glov_3695 | Hypothetical | 936 | 0.92 |
| Glov_3696 | SAM domain integrase | 1014 | 1.03 |
| Glov_3697 | Hypothetical, No NCBI database hits | 231 | 1.01 |
| Glov_3698 | Transposase Tn3 | 2925 | 1.00 |
| Glov_3700 | NADPH-dependent FMN reductase | 504 | 0.94 |
| Glov_3701 | Alkylhydroperoxidase | 456 | 1.08 |
| Glov_3702 | Hypothetical, No NCBI database hits | 276 | 0.86 |
| Glov_3703 | Hypothetical | 408 | 0.87 |
| Glov_3704 | TetR family transcriptional regulator | 573 | 1.04 |
| Glov_3705 | Hypothetical, No NCBI database hits | 336 | 0.91 |
| Glov_3706 | MTH865-like protein | 237 | 1.01 |
| Glov_3707 | NADPH-dependent FMN reductase | 627 | 0.95 |
| Glov_3708 | Tautomerase | 399 | 1.06 |
| Glov_3709 | Integrase | 978 | 1.05 |
| Glov_3710 | Hypothetical | 588 | 0.92 |
| Glov_3711 | DSBA oxidoreductase | 588 | 1.00 |
| Glov_3712 | Short-chain dehydrogenase/reductase | 735 | 0.96 |
| Glov_3713 | IS3/IS911 transposase | 309 | 0.90 |
| Glov_3714 | Integrase | 861 | 0.92 |
| Glov_3715 | IstB domain protein | 762 | 1.14 |
| Glov_3716 | Integrase | 1572 | 1.13 |
| Glov_3717 | LysR family transcriptional regulator | 867 | 1.00 |
| Glov_3718 | Cob(I)alamin adenosyl-transferase, cobA/btuR | 663 | 1.00 |
| Glov_3719 | 2-Oxoglutarate-ferredoxin-oxidoreductase, beta subunit | 1002 | 1.06 |

**Table S9.** (Continued)

| pSZ77 locus | Inferred/Annotated Function | Length (bp) | Normalized CAI |
| --- | --- | --- | --- |
| Glov_3720 | 2-oxoglutarate-ferredoxin-oxidoreductase, alpha subunit | 1854 | 1.08 |
| Glov_3721 | Vitamin B12-independent methionine synthase | 1098 | 0.97 |
| Glov_3722 | Hypothetical | 291 | 0.90 |
| Glov_3723 | Ribosomal small subunit-dependent GTPase A | 1002 | 1.01 |
| Glov_3724 | NADPH-dependent FMN reductase | 642 | 0.92 |
| Glov_3725 | Crp/FNR family transcriptional regulator | 714 | 1.03 |
| Glov_3726 | Haloacid dehalogenase, type II ** | 687 | 1.00 |
| * Normalized to codon usage over the entire strain SZ chromosome  ** The function of the pSZ77-encoded haloacid dehalogenase is unknown but the protein shares 31% identity (51% similarity) with the characterized 2,2-dichloropropanoic acid dehalogenase from *Pseudomonas putida* strain PP3 [82]. | | | |
