# Supplementary material for: Genomic determinants of organohalide-respiration in Geobacter lovleyi, an unusual member of the Geobacteraceae
Source: BMC Genomics. 2012 May 22;13:200. doi: 10.1186/1471-2164-13-200 (PMC3403914; doi:10.1186/1471-2164-13-200)
Supplement: Additional file 13 — DNA sequence differences between the pSZ77 and the KB-1 plasmid assemblies. [file 1471-2164-13-200-S13.doc]

**Additional file 13:**  DNA sequence differences between the pSZ77 and the KB-1 plasmid assemblies.

| pSZ77 locus | Function | Deletions from KB-1 plasmid |
| --- | --- | --- |
| Glov_3646 | Precorrin-6y methyltransferase | 1 bp deletion 195 bp after start disrupts reading frame on KB-1 |
| Glov_3654 | Precorrin-8X methylmutase | 136 bp deletion 361 bp from start codon |
| Glov_3676 to Glov_3675 | Intergenic region | 4 bp deletion 26 bp upstream of Glov_3675 start  1 bp deletion 10 bp upstream of Glov_3675 start |
| Glov_3690 | HipA | 1 bp deletion 717 bp after Glov_3690 start and  81 bp deletion 726 bp after Glov_3690 start, both disrupt reading frame on KB-1 |
|  |  |  |
| pSZ77 locus | Function | Insertions on KB-1 plasmid not found on pSZ77 |
| Glov_3700 | NADPH-dependent FMN reductase | 119 bp insertion at 5’ end of region corresponding to Glov_3699 pseudogene |
| Glov_3701 | Alkylhydroperoxidase | A 123 bp insertion 80 bp after start of Glov_3701 disrupts reading frame on KB-1 |
| Glov_3712 to Glov_3713 intergenic | Transposase IS3/IS911 | A 136 bp insertion 61 bp upstream of Glov_3713 |
|  |  |  |
| pSZ77 locus | Function | Single Nucleotide Polymorphisms (SNPs) |
| Glov_3702 | Hypothetical, no prokaryotic BlastP hits | T 🡪 G changes stop codon to glutamic acid on KB-1 plasmid |
| Glov_3709 | Integrase | Synonymous G 🡪 A 360 bp after start codon |
