# Supplementary material for: Genomic determinants of organohalide-respiration in Geobacter lovleyi, an unusual member of the Geobacteraceae
Source: BMC Genomics. 2012 May 22;13:200. doi: 10.1186/1471-2164-13-200 (PMC3403914; doi:10.1186/1471-2164-13-200)
Supplement: Additional file 14 — Detection of the pSZ77repAgene andGeobacteraceaespp. 16S rRNA genes in pure and mixed cultures containing Geobacter strains with specific PCR primers. Top row: repA gene-targeted PCR (565 bp amplicon) and bottom row Geobacteraceae 16S rRNA gene-targeted PCR (312 bp amplicon). The arrows indicate the expected PCR amplicons. Lane 1: 1 kb Plus DNA ladder (Invitrogen), Lane 2: no template (negative control), Lane 3: G. lovleyi strain SZ (positive control), Lanes 4 and 5: consortium BDI, Lane 6: consortium KB-1, Lane 7: Ft. Lewis isolate 7.1, Lane 8: Ft. Lewis isolate 7.2, Lane 9: Ft. Lewis isolate 7.3, Lane 10: Ft. Lewis isolate 7.4, Lane 11: G. thiogenes. [file 1471-2164-13-200-S14.doc]

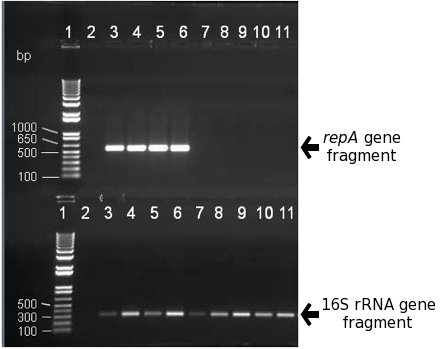


**Additional file 14:** Detection of the pSZ77 *repA* gene and *Geobacteraceae* spp. 16S rRNA genes in pure and mixed cultures containing *Geobacter* strains with specific PCR primers. Top row: *repA* gene-targeted PCR (565 bp amplicon) and bottom row *Geobacteraceae* 16S rRNA gene-targeted PCR (312 bp amplicon). The arrows indicate the expected PCR amplicons. Lane 1: 1 kb Plus DNA ladder (Invitrogen), Lane 2: no template (negative control), Lane 3: *G. lovleyi* strain SZ (positive control), Lanes 4 and 5: consortium BDI, Lane 6: consortium KB-1, Lane 7: Ft. Lewis isolate 7.1, Lane 8: Ft. Lewis isolate 7.2, Lane 9: Ft. Lewis isolate 7.3, Lane 10: Ft. Lewis isolate 7.4, Lane 11: *G. thiogenes*.
